# Aryl-, Akynyl-, and Alkenylbenziodoxoles: Synthesis and Synthetic Applications

**DOI:** 10.3390/molecules28052136

**Published:** 2023-02-24

**Authors:** Irina A. Mironova, Dmitrii M. Noskov, Akira Yoshimura, Mekhman S. Yusubov, Viktor V. Zhdankin

**Affiliations:** 1 Research School of Chemistry and Applied Biomedical Sciences, Tomsk Polytechnic University, 634050 Tomsk, Russia; 2 Faculty of Pharmaceutical Sciences, Aomori University, 2-3-1 Kobata, Aomori 030-0943, Japan; 3 Department of Chemistry and Biochemistry, University of Minnesota Duluth, Duluth, MN 55812, USA

**Keywords:** hypervalent iodine, functionalization, benziodoxoles, arylation, benzyne, alkynylation, vinylation, EBX, VBX

## Abstract

Hypervalent iodine reagents are in high current demand due to their exceptional reactivity in oxidative transformations, as well as in diverse umpolung functionalization reactions. Cyclic hypervalent iodine compounds, known under the general name of benziodoxoles, possess improved thermal stability and synthetic versatility in comparison with their acyclic analogs. Aryl-, alkenyl-, and alkynylbenziodoxoles have recently received wide synthetic applications as efficient reagents for direct arylation, alkenylation, and alkynylation under mild reaction conditions, including transition metal-free conditions as well as photoredox and transition metal catalysis. Using these reagents, a plethora of valuable, hard-to-reach, and structurally diverse complex products can be synthesized by convenient procedures. The review covers the main aspects of the chemistry of benziodoxole-based aryl-, alkynyl-, and alkenyl- transfer reagents, including preparation and synthetic applications.

## 1. Introduction

In the past decades, hypervalent iodine chemistry has attracted the active interest of organic chemists all over the world due to the versatile and ecologically benign nature of hypervalent iodine reagents [1,2,3,4,5]. Five-membered cyclic iodine compounds, known under the general name of ‘benziodoxoles’, are particularly important as reagents because they have considerably higher thermal stability compared to their acyclic analogs [6,7]. This stabilization is usually explained by the lower reactivity of the hypervalent iodine center toward reductive elimination because of the link between apical and equatorial positions via the five-membered ring, as well by a better overlap of the lone pair electrons on the iodine atom with the π orbitals of the benzene ring [6]. Despite the higher thermal stability, some benziodoxoles, such as azidobenziodoxoles, are high-energy compounds that in some cases are prone to explosive degradation and should be manipulated with adequate precautions [7,8,9]. Benziodoxoles are widely utilized in organic synthesis as the umpolung iodine(III) reagents for introducing various functional groups, such as alkynyl, alkenyl, CN, SCN, N_3_, CF_3_, Hal, etc., and are generally named as ‘atom-transfer reagents’ [7,8,9,10,11].

**Figure 1:**
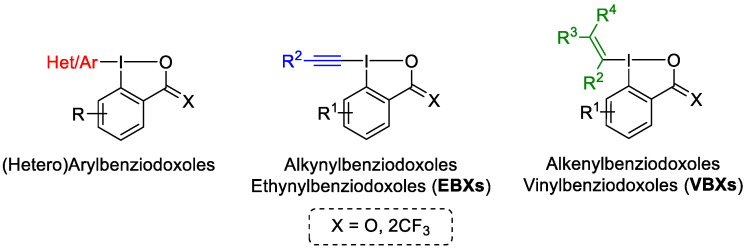
Arylbenziodoxoles, ethynylbenziodoxoles, and vinylbenziodoxoles.

The synthetically important C-functionalization (arylation, alkenylation, and alkynylation) of organic molecules is usually achieved by coupling reactions requiring the use of transition metals, pre-functionalized substrates, and other expensive or hard-to-get reagents [12,13,14,15,16]. Benziodoxole reagents that contain carbon-based functional groups at the iodine(III) center (aryl-, alkynyl-, and alkenylbenziodoxoles, Figure 1) allow carrying out C-C and C-heteroatom bond-forming reactions under transition metal-free conditions, or under mild and easy-to-handle catalytic conditions. In the current review, we discuss preparation, structural aspects, and recent synthetic applications of aryl-, alkynyl-, and alkenylbenziodoxoles.

## 2. Arylbenziodoxoles

### 2.1. Synthesis and Structure

**Scheme 1:**
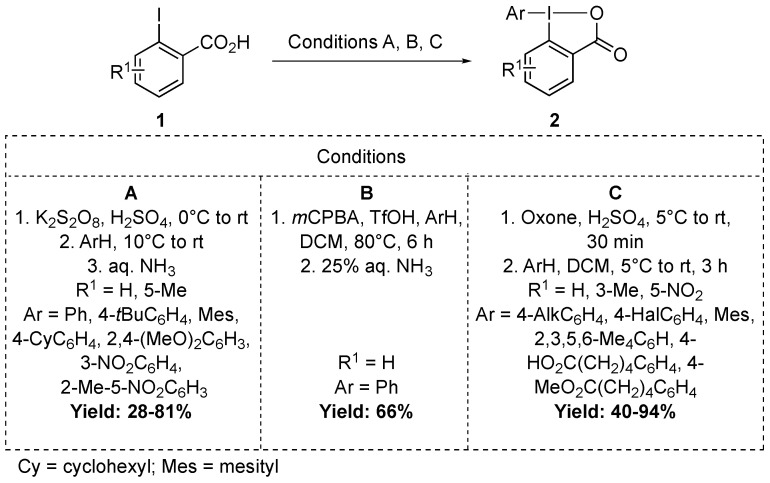
Methods of synthesis of arylbenziodoxoles **2** under conditions **A**–**C**.

Arylbenziodoxoles can be formally considered as internal iodonium salts bearing the anionic carboxylate moiety in the *ortho* position to the aryliodonium group. Phenylbenziodoxole [also known as diphenyliodonium-2-carboxylate, **2** (R^1^ = H, Ar = Ph)] is the most known and commercially available representative of arylbenziodoxoles. Various substituted arylbenziodoxoles **2** can be conveniently prepared from 2-iodobenzoic acids **1** and the corresponding substituted benzenes under different conditions (**A**, **B**, or **C**, Scheme 1), using potassium persulfate [17,18,19,20,21], *m*-CPBA [22], and oxone [23,24]. The last approach is the most convenient one-pot procedure for the synthesis of various substituted arylbenziodoxoles **2** with yields up to 94%, using oxone as an inexpensive and environmentally safe oxidant in the presence of sulfuric acid (Scheme 1, conditions C) [23].

**Scheme 2:**
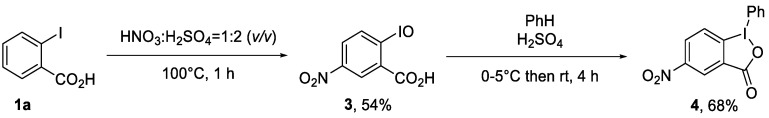
Preparation of 5-nitro-1-phenyl-1λ^3^-benzo[*d*] [1,2]iodaoxol-3(1*H*)-one **4**.

Nitro-substituted phenylbenziodoxole **4** also can be prepared in two steps from 2-iodobenzoic acid **1**a by oxidation nitration using the mixture of fuming nitric and concentrated sulfuric acid and followed by treatment with benzene in concentrated sulfuric acid (Scheme 2) [18,25].

**Scheme 3:**
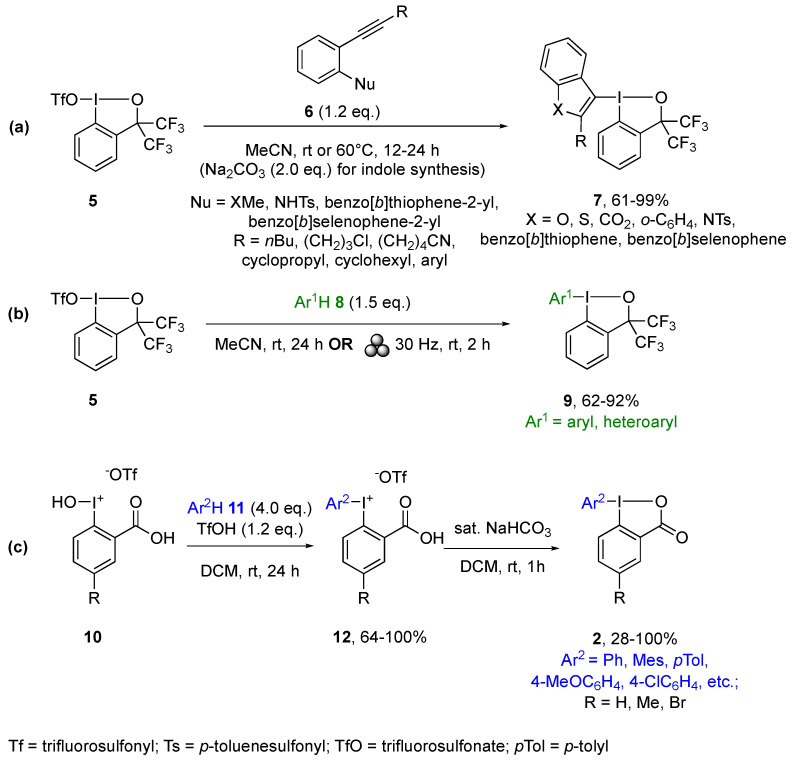
Synthesis of various (hetero)arylbenziodoxoles **7, 9,** and **2** from hypervalent iodine reagents **5** and **10,** respectively. See explanation of reactions (**a**–**c**) in the text.

Later, two different scientific groups independently proposed the method of preparation of various arylbenziodoxoles **7, 9,** and **2** from hypervalent iodine reagents **5** and **10,** respectively (Scheme 3). Yoshikai and co-workers used benziodoxole triflate **5** as a versatile reagent for iodo(III)cyclization of alkynes **6** that afforded various air and thermally stable (hetero)aryl-λ^3^-iodanes **7** under simple and mild conditions (Scheme 3a) [26]. While aryl-λ^3^-iodanes are typically synthesized by oxidation of iodoarenes or exchange with organometallic compounds, this cyclization offers unique access to a wide variety of (hetero)arylbenziodoxoles **7**, bearing benzofurans, benzothiophenes, isocoumarins, indoles, and polyaromatics.

In 2020, the same group started from bis(trifluoromethyl)benziodoxole triflate **5** using various arenes **8** to obtain different aryl- and (hetero)arylbenziodoxoles **9** with high yields (Scheme 3b) [27]. In addition to the broad scope of arenes **8**, this method could be performed under solvent-free conditions. It should be noted that this method has important limitations: the less electron-rich arenes require the use of Lewis acid as a catalyst; arylbenziodoxoles with electron-neutral or electron-poor aryl moiety could be prepared in good yields via silicon–iodine(III) or boron–iodine(III) aryl transfer reactions using corresponding aryltrimethylsilanes or aryltrifluoroborates, whereas the original conditions did not afford desired products **9** with initial benziodoxole **5** even at increased temperatures (up to 80 °C) [27].

Later, IBA-TfOH **10** (IBA = 2-iodosylbenzoic acid) and arenes **11** have been used as starting materials for the preparation of pseudocyclic arylbenziodoxole triflates **12** in the first step followed by treatment of compounds **12** with a saturated NaHCO_3_ solution at room temperature to afford desired arylbenziodoxoles **2,** with the yields up to 100% (Scheme 3c) [28].

**Scheme 4:**
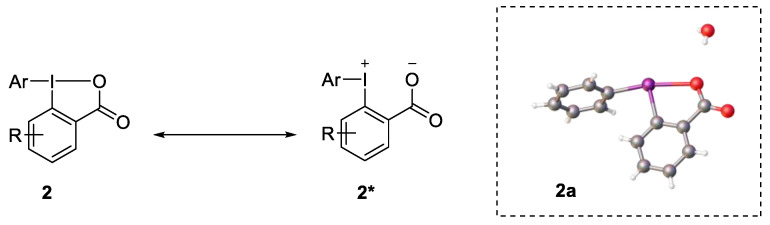
Resonance structure 2* of arylbenziodoxoles **2** and X-ray structure of **2a**.

Single-crystal X-ray structures have been published for several arylbenziodoxoles **2** [23,29,30,31]. According to single-crystal X-ray diffraction data, arylbenziodoxoles **2** have a zwitterionic structure characterized by the presence of a short internal I···O interaction [23,24,25,26,27,28,29]. The average distance of the intramolecular I···O bonds (2.5 Å) is longer than the average covalent I-O bond length (2.14 Å) [32,33] but shorter than the sum of van der Waals radii for iodine atom and an oxygen atom (3.5 Å) [34], which is indicative of a significant increase in the ionic nature of this bond. The structure of arylbenziodoxoles **2** can be described using resonance contributors **2** and 2* (Scheme 4) [23,24,25,26,27,28,29]. The zwitterionic character of arylbenziodoxoles (resonance contributor 2*) is reflected in their common name of diaryliodonium-2-carboxylates. In general, benziodoxoles have a planar structure with a highly distorted T-shaped geometry around iodine. The observed bond angle C−I−O in benziodoxoles is about 80°, which is significantly different from the 90° angle typical of noncyclic hypervalent iodine compounds [1,2].

### 2.2. Synthetic Applications

#### 2.2.1. Benzyne Generation by Thermal Decomposition

Arylbenziodoxoles, in particular phenylbenziodoxole **2a**, are common benzyne precursors [35]. Aryne generation from precursors **2** by thermal decomposition was investigated extensively in the 20th century and summarized in earlier reviews [1,2,35]; therefore, only a brief discussion of these reactions will be provided in this section.

**Scheme 5:**
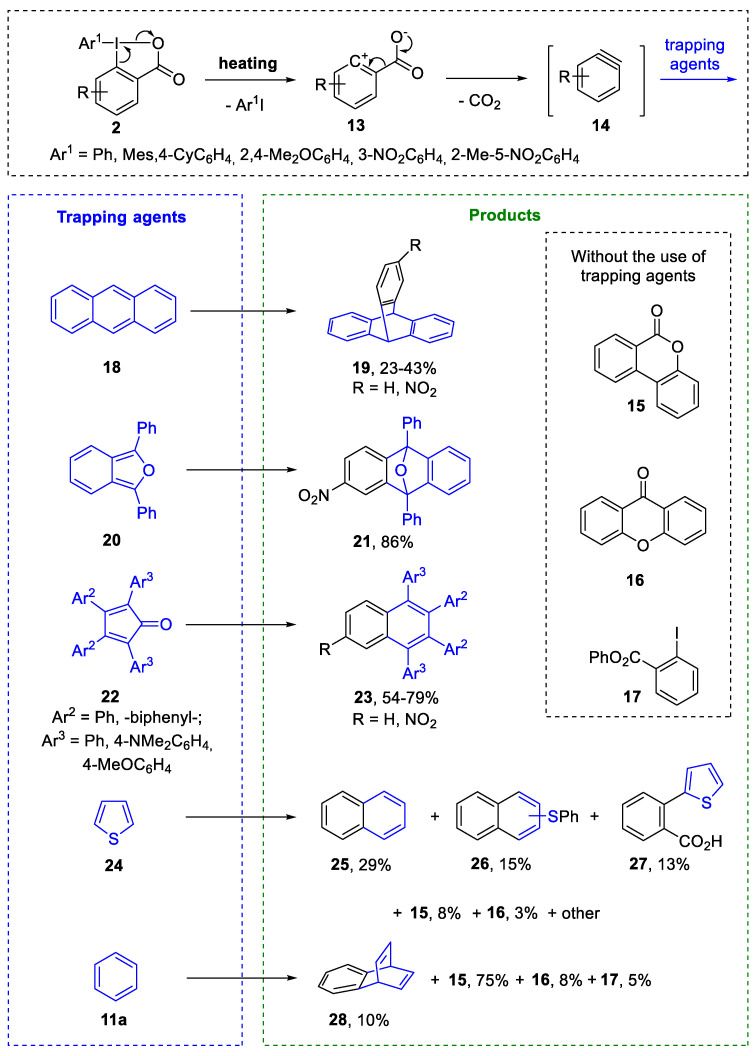
Thermolysis of arylbenziodoxoles **2** in the absence and the presence of aryne-trapping agents **18**, **20**, **22**, **24**, and **11a**.

Arylbenziodoxoles **2** have been employed as effective aryne **14** sources in the presence of common benzyne trapping compounds such as anthracene **18**, 1,3-diphenylisobenzofuran **20**, and tetracyclone compounds **22,** with the formation of products **19**, **21,** and **23,** respectively (Scheme 5) [18,19,25,36,37,38,39]. It was shown the efficiency of benzyne trapping reagents increases in the order: anthracene **18** < 1,3-diphenylisobenzofuran **20** < 2,3,4,5-tetraphenylcyclopentadienone (tetracyclone) **22a** < 2,5-bis(*p*-dimethylaminopheny1)-3,4-diphenylcyclopentadienone **22b** < 2,5-di-*p*-anisyl-3,4-diphenylcyclopentadienone **22c** [18].

Thiophenes **24** and benzene **11a** have been found to react with benzyne species **14** to give a mixture of cycloaddition products, such as naphthalene **25,** α- and β-naphthylphenylsulfides **26**, 2-(2-thienyl)carboxylic acid **27,** and benzobarrelene **28,** respectively [40,41,42,43,44,45], in addition to the known decomposition benzyne adducts **15–17** (Scheme 5) [18,38,46].

**Scheme 6:**
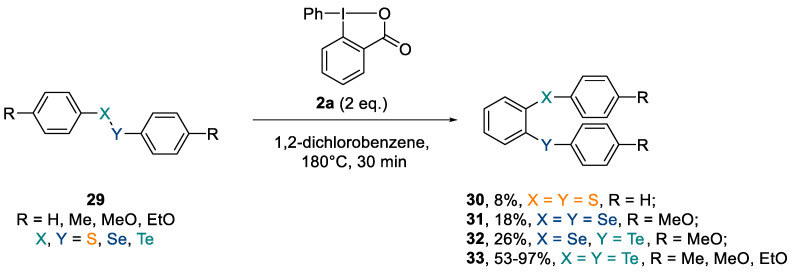
Reactions of **2a** with diarylchalcogenides **29** under reflux conditions.

1-Phenylbenziodoxole **2a** is a particularly useful reagent for the reactions leading to new carbon–heteroatom bond formation. The reaction of **2a** using diaryldichalcogen compounds (S, Se, Te) **29** at reflux conditions afforded the respective *ortho*-hetero-disubstituted phenylene compounds **30–33** in low to high yields [20,46,47]. Similarly, the treatment of bis(4-methoxyphenyl)selenaditelluride **29a** with **2a** yielded the selenatelluride compound **32** in 26% yield (Scheme 6).

**Scheme 7:**
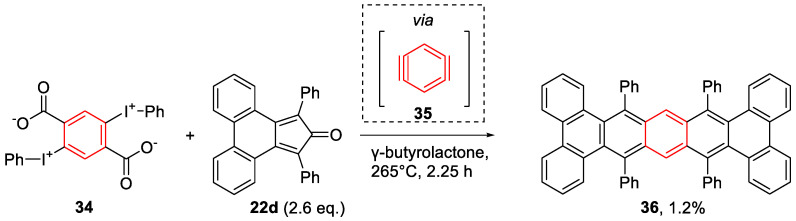
Synthesis of polycyclic compound **36** with the use of bisiodonium dicarboxylate **34**.

The 1,4-benzadiyne species **35** can be generated from 1,4-bis(phenyliodonio)benzene-2,5-dicarboxylate **34** under reflux conditions and trapped by the reaction with phencyclone **22d** to give polycyclic aromatic hydrocarbon, 9,11,20,22-tetraphenyltetrabenzo[*a,c,l,n*]pentacene **36** (Scheme 7) [48]. The precursor **34** of 1,4-benzadiyne was prepared from 2,5-diiodoterephalic acid by using the original synthetic methodology reported by Beringer [17].

#### 2.2.2. Nucleophilic Substitution

**Scheme 8:**
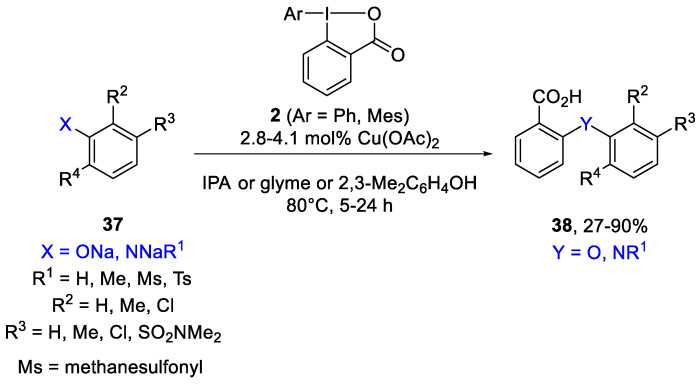
Reactions of **2** with nucleophiles **37** under copper(II) catalysis.

Contrary to benzyne formation and its trapping reactions, it is possible to use arylbenziodoxoles in reaction with nucleophiles to afford benzoic acid derivatives. In such a way, Scherrer and Beatty first proposed copper-catalyzed condensation of arylbenziodoxoles **2** with N- and O-nucleophiles **37** to give *ortho*-substituted benzoic acids **38** (Scheme 8) [49].

**Scheme 9:**
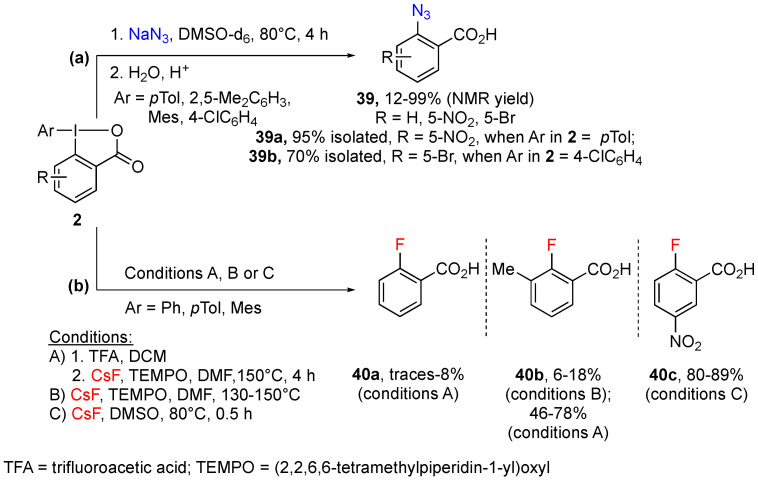
Reactions of **2** with N_3_^-^ (reaction (**a**)) and F^-^ (reaction (**b**)) under metal-free conditions **A**–**C**.

The reactivity study of arylbenziodoxoles **2** in reactions with nucleophiles revealed that *ortho*-methyl-substituted benziodoxoles, such as 1-phenyl-7-methylbenzidoxole **2b**, are more reactive than 1-phenylbenziodoxole **2a** [23]. This enhanced reactivity of 1-phenyl-7-methylbenzidoxole **2b** was explained by the steric effect of *ortho*-substituent on the nucleophillic substitution in diaryliodonium salts. Later, the same group continued the study of nucleophilic substitution of the iodonium leaving the group in arylbenzoiodoxoles **2** with azide anion to afford 2-azidobenzoic acids **39** (Scheme 9a) [24,28]. The presence of bulky substituents in the *ortho* position of the aryl ring slows the reaction down, while the presence of a moderately electron-withdrawing bromine substituent in the *para* position to the iodine atom in the benziodoxolone ring moderately increases the rate of substitution. The presence of a strongly electron-withdrawing nitro group in the *para* position to the iodine atom in the benziodoxolone ring dramatically increases the rate of substitution. These observations are in agreement with the electronic requirements for internal nucleophilic substitution in the benziodoxole ring.

The same group has shown the possibility of using arylbenziodoxoles **2** as efficient precursors for the synthesis of fluorobenzoic acids **40** via nucleophilic fluorination using fluoride salts in polar aprotic solvents (Scheme 9b) [50]. In particular, 5-nitro-substituted benziodoxole **2c** was found to be an excellent reagent for the radiofuorination leading to [^18^F]-fluorobenzoic acids in up to 39% of the radiochemical yield, with excellent radiochemical purity above 98%. This protocol under optimized reaction conditions (30 min at 150 °C in acetonitrile) was applied for the preparation of 2-[^18^F]-fluoro-5-nitrobenzoic acid **40c**, which is a potentially important radioligand for positron emission tomography (PET) [51,52].

**Scheme 10:**
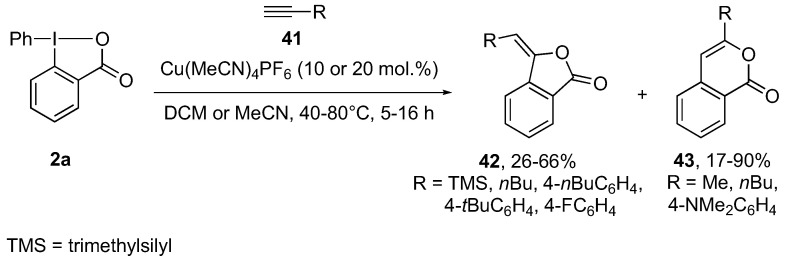
Pd-free Sonogashira-like coupling reaction with the use of 1-phenylbenziodoxole **2a** in the synthesis of phthalides **42** and isocoumarins **43**.

The first example of a Pd-free Sonogashira-like coupling reaction of acetylenes **41** with 1-phenylbenziodoxoles **2a** in the synthesis of phthalides **42** and isocoumarins **43** under Cu^I^ catalysis in the absence of bases was reported (Scheme 10) [31]. High selectivity and yields were achieved under mild reaction conditions with good functional group tolerance.

**Scheme 11:**
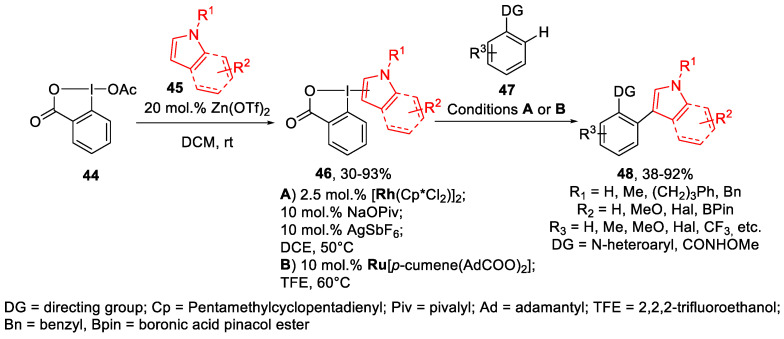
Rh- and Ru-catalyzed C-H functionalization of arenes using indole- and pyrrole-benziodoxoles **46** under reaction conditions **A** or **B**.

In 2017, Waser and co-workers developed the method of synthesis of various 1-heteroarylbenziodoxoles **46** from acetoxybenziodoxole **44** and indoles or pyrroles **45** in one-step under mild Lewis acid catalyzed conditions (Scheme 11) [53]. Furthermore, they proposed the *ortho* C-H functionalization method of unactivated arenes **47** with the use of indole- and pyrrole-benziodoxoles **46** under either rhodium or ruthenium catalysis to afford a broad range of heterocyclic systems **48** of high interest for synthetic and medicinal chemistry [53].

## 3. Ethynylbenziodoxoles (EBXs)

### 3.1. Synthesis and Structure

Alkynylbenziodoxoles also named ethynylbenziodoxoles (EBXs) emerged as alkyne-transfer reagents at the beginning of the 21st century. Umpolung reactions with the use of hypervalent iodine reagents in particular EBXs have been developing because of the need for efficient and flexible methods of introduction of various functional groups in different sites of a molecule. Acetylenes have always been one of the most important and versatile functional groups in organic chemistry, as well as a tool and a structural element in material science and chemical biology [54]. EBX reagents can be effectively applied as electrophilic alkynylating reagents to various organic nucleophiles; moreover, their utilization is often preferable in contrast to classical methods [7,9,11,55,56,57,58,59,60].

**Scheme 12:**
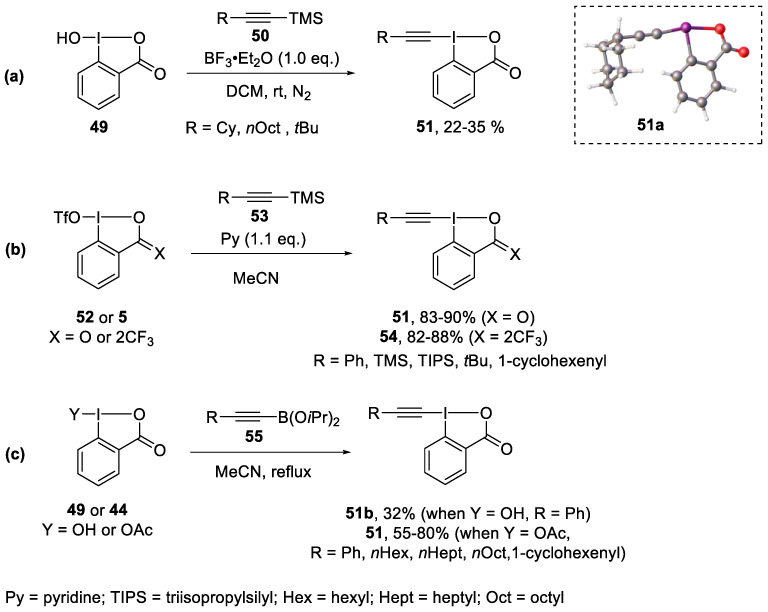
Synthesis of alkynylbeziodoxoles **51** and **54** from hypervalent iodine(III) reagents **5**, **44**, **49,** and **52** and X-ray structure of **51a**. See explanation of reactions (**a**–**c**) in the text.

The first example of EBX was prepared in 1991 by treating IBA **49** with 1-alkynyltrimethylsilane **50** in anhydrous dichloromethane at ambient temperature in the presence of BF_3_∙Et_2_O followed by heating in methanol at 60 °C. (Scheme 12a) [61]. The same group proved the structure of cyclohexyl-EBX **51a** by X-ray diffraction analysis (Scheme 12a) [61]. X-ray structural data revealed a distorted T-shaped geometry expected for hypervalent iodine with an endocyclic C(sp^2^)-I-O angle of 75.28° and a C(sp^2^)-I-C(sp) angle of 90.9°. The lengths of the bonds to the iodine atom, I-C(sp^2^) (2.14 Å), I-O (2.34 Å), and I-C(sp) (2.03 Å), are within the range of typical single covalent bond lengths in noncyclic organic derivatives of polyvalent iodine. In the next decades, many X-ray structures of various aromatic and alkylic alkynylbenziodoxoles, as well as silyl alkynylbenziodoxoles, were obtained with almost the same bond lengths and angles at the iodine atom [62].

An improved procedure for the preparation of various alkynylbenziodoxoles **51** and **54** in high yields involves the reaction of triflates **52** or **5** with alkynyltrimethylsilanes **53** (Scheme 12b) [63].

In 2000, compound **51b** was obtained by treating 2-iodosylbenzoic acid **49** with alkynylboronate **55** (Scheme 12c); however, with a lower yield [64]. The low yield in this reaction can be explained by the low solubility of benziodoxole **49** in organic solvents and its acidic properties (pKa 7.25). The relatively high acidity of benziodoxole **49** can lead to the decomposition of the alkynylboronates **55** under the reaction conditions. The replacement of starting compound **49** with acetoxybenzoiodoxole **44** improved the yield of EBXs **51,** as well as shortened the reaction time from 20 to 6 h (Scheme 12c) [64].

In 2010, Brand and Waser slightly modified the method [62] by using bis-silylated alkynes in combination with TMSOTf [65]. Synthesized TIPS-EBX has been employed for the alkynylation of thiophenes, which process is considered in Section 3.2.1. This method [66] was used in the synthesis of various alkynylbenziodoxoles with minor changes in many others studies [62,66,67,68,69,70,71,72,73,74,75,76,77,78,79].

**Scheme 13:**
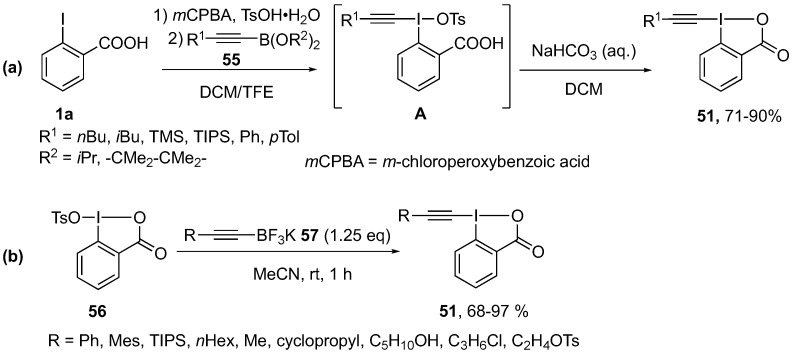
One-pot syntheses of alkynylbenziodoxoles **51** via intermediate **A**. See explanation of reactions (**a**,**b**) in the text.

Later, Olofsson and co-workers proposed a one-pot method for the synthesis of alkynylbenziodoxoles **51** from 2-iodobenzoic **1a** acid using *m*CPBA and *p*TsOH for hypervalent iodine species formation, followed by the addition of alkynylboronates **55** at ambient temperature to afford alkynyliodonium tosylate **A** (Scheme 13a) [80]. However, this method requires isolation and purification of the reagent for each modification. This method was modified in 2019 by replacing the boronic ethers with trimethylsilyltriisopropylsilyl acetylene; however, the yields have slightly decreased [81]. Authors mention that TIPS-EBX obtained by using the latter protocol is not shock-sensitive and has the same thermal stability as when accessed using previous methods, but tosylate impurities have to be carefully removed, as they lead to lower decomposition temperatures.

Very recently, Waser et al. utilized tosylate **56** and alkynyltrifluoroborates **57** for rapid and highly effective formation of EBX reagents **51** without the use of any additives (Scheme 13b) [82]. The EBXs **51** obtained in this way did not require the use of column chromatography for purification.

**Scheme 14:**
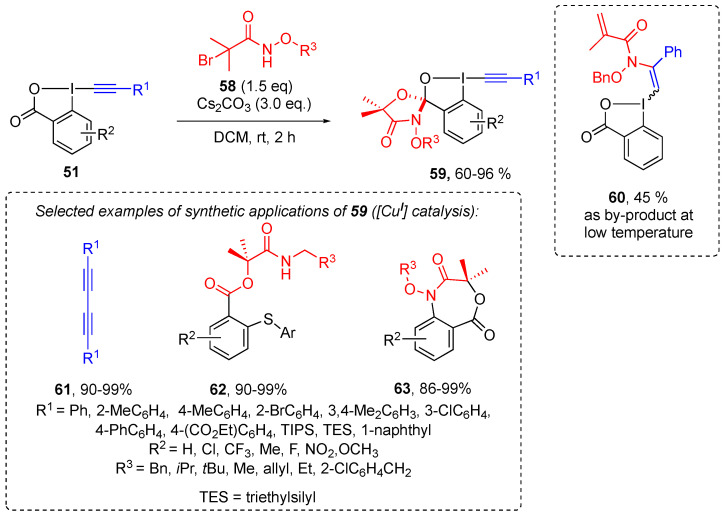
Synthesis of spirocyclic alkynylbenziodoxoles **59** and products **61**–**63** of their synthetic applications.

The first representatives of spirocyclic alkynylbenziodoxoles **59** were prepared by exposure to EBXs **51** of α-bromoamide **58** under basic conditions at room temperature (Scheme 14) [83,84]. Vinylbenziodoxole (VBX) **60** has been formed as a by-product during the reaction course, but at low temperatures its formation was maximal and, finally, compound **60** was isolated, with a yield of 45%. Spirocyclic EBXs **59** have been employed in the synthesis of 1,3-diynes **61** [83,84], diaryl thioethers **62** [84], and 4,1-benzoxazepine-2,5-diones **63** [83] under copper(I) catalysis.

### 3.2. Synthetic Applications

Several reviews on the utilization of EBXs in direct alkynylation processes or complex reactions with the formation of several bonds in a single transformation were previously published [7,9,11,55,56,57,58,59,60,85]. In this section, we summarize general procedures of alkynylations with EBXs and overview the most significant recent works. The reactions of EBXs as Michael acceptors with the formation of vinylbenziodoxoles (VBXs) are discussed in Section 4.

#### 3.2.1. Metal-Catalyzed Alkynylation Reactions

##### Gold Catalysis

**Scheme 15:**
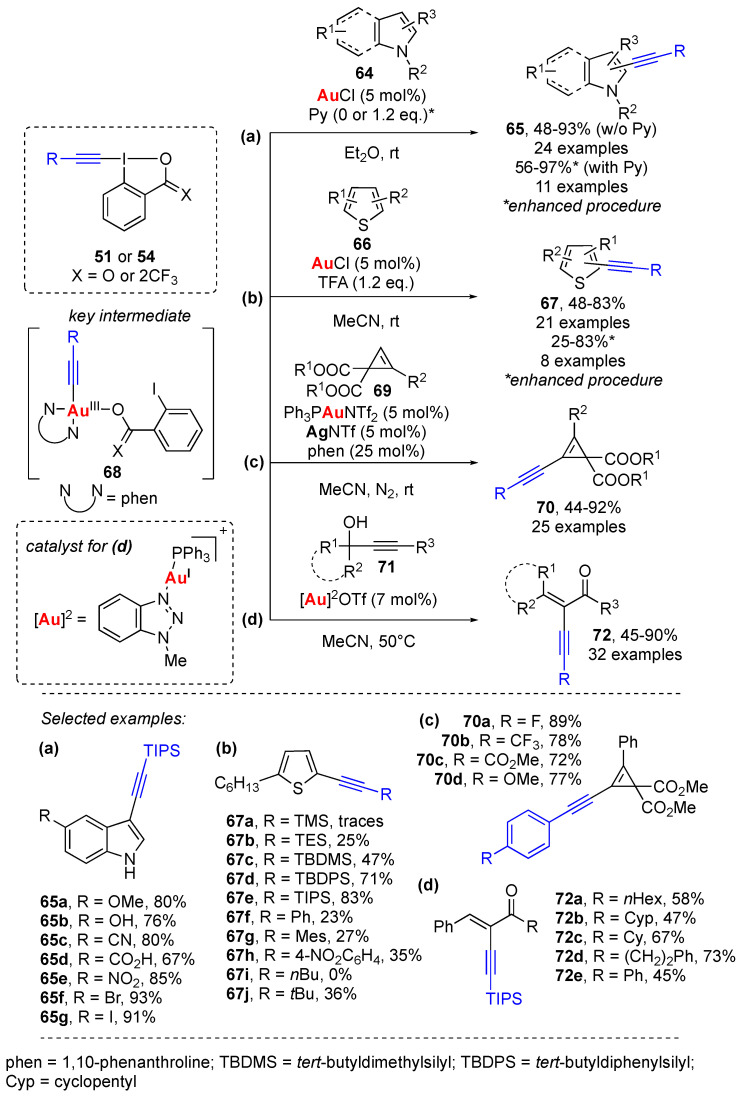
Gold-catalyzed alkynylation reactions with the use of EBXs **51** and **54**. See explanation of reactions (**a**–**d**) in the text. Selected examples of products obtained in the reactions (**a**–**c**) are shown at the bottom of the scheme.

The first gold-catalyzed direct alkynylation of indole and pyrrole heterocycles **64** using EBXs **51** was reported by Waser and co-workers in 2009 (Scheme 15a) [86]. Later, the same group proposed direct alkynylation of thiophenes **66** using a modified method (Scheme 15b) [65]. In their next work, the procedure was improved, and the scope of utilized EBXs and substrates was extended to afford various alkynes **65** and **67** (Scheme 15a,b) [62]. Bulky silyl groups as alkyne substituents were found to be optimal and the transfer of aromatic acetylenes to thiophene **66** was achieved for the first time. Control reactions between substrates of different nucleophilicity and deuterium labeling experiments, as well as the regioselectivity observed, were all in agreement with electrophilic aromatic substitution. Investigations indicated that gold(III) could be eventually reduced to gold(I) during the process, and based on the results of this mechanistic study, the authors assumed a π activation or an oxidative mechanism was the most probable for the alkynylation reaction [62]. However, Ariafard et al. reported computational results that both the oxidative and the π activation mechanisms were too high in energy and suggested that the iodine(III) center in EBXs acts as a Lewis acid for activating the alkyne even more efficiently than the Au(I)-center [87]. In 2019, Hashmi’s group reported an investigation of the oxidative process that involves a tri- or tetra-coordinate Au(I) intermediate with an oxidizing agent, particularly EBX, and provided strong experimental and computational evidence in favor of the oxidative addition of EBX to the tri-coordinate (phen)Au^I^L species to generate **68** (Scheme 15, key intermediate) [88,89]. The review [55] was also dedicated to the rationalization of gold-catalyzed alkynylation, proposing a probable ‘interplay mode’ wherein Au-catalysts activate the π system embedded in the partner nucleophile and are also oxidized to Au(III) by EBXs with the formation of intermediate **68**. Common to all proposed mechanistic pathways is an electrophilic aromatic substitution step, which explains the high regioselectivity observed.

A gold-catalyzed direct alkynylation of cyclopropenes **69** with EBXs **54** is enabled by two operating catalytic cycles, an oxidative catalytic cycle involving an alkynyl Au(III) complex **68** formed by oxidative addition and the second one involving a silver-mediated C-H activation (Scheme 15c) [88]. As a result, a wide range of functionalized cyclopropenes **70** was obtained with moderate to excellent yields.

The first alkynylative Meyer–Schuster rearrangement, which was previously unsuccessful under Pd catalysis [90], was developed by harnessing the potential of the ‘interplay mode’ of gold catalysis, which integrates the π activation mode and an EBX-enabled cross-coupling mode (Scheme 15d) [91]. The reaction offers straightforward access to diverse (*E*)-enynones **72** from alkynols **71**, barring the formation of any undesired enone side products.

**Scheme 16:**
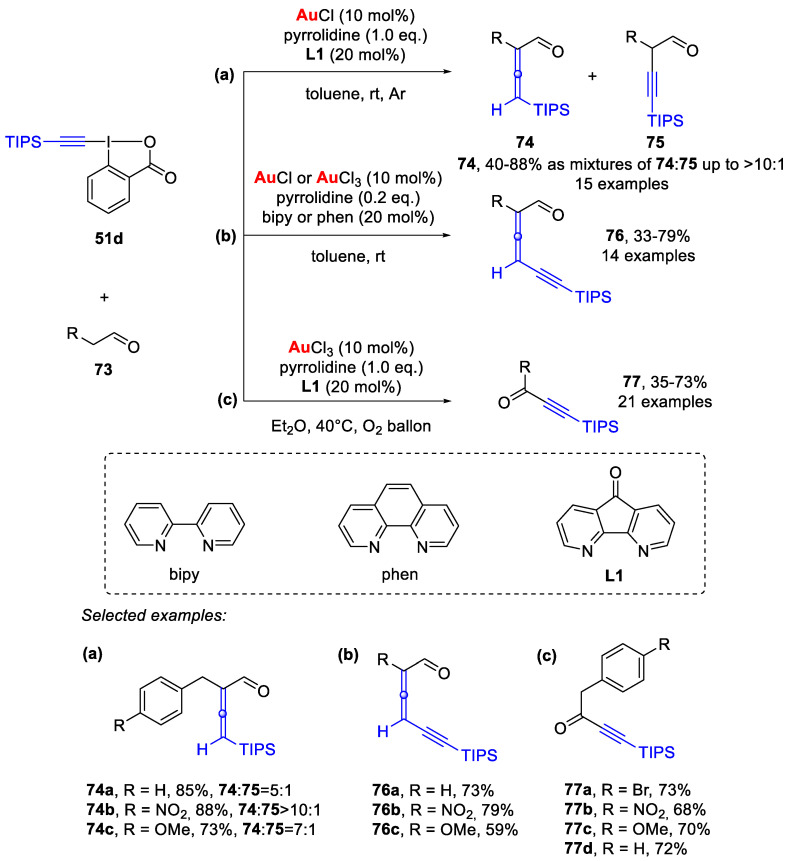
Gold/pyrrolidine catalyzed α-vinylidenation reaction of aldehydes **73** with the use of TIPS-EBX **51d**. See explanation of reactions (**a**–**c**) in the text. Selected examples of products obtained in the reactions (**a**–**c**) are shown at the bottom of the scheme.

The first direct α-vinylidenation (Scheme 16a) with the formation of both formyl allenes **74** and alkynylated aldehydes **75**, and the α-vinylidenation/γ-alkynylation cascade of aldehydes **73** (Scheme 16b) using TIPS-EBX **51d** with a synergistic gold/amine catalyst system, was reported by Huang’s group [92]. Functionality rich, tri-, and tetra-substituted allenes **76** bearing a versatile aldehyde and an acetylene functionality were prepared in a straightforward protocol. Later, the same group developed a direct synthesis of diverse ynones **77** from readily available aldehydes **73** and TIPS-EBX **51d** under gold/pyrrolidine synergistic catalysis (Scheme 16c) [93]. The reaction proceeds through the α-vinylidenation reaction, followed by the in situ C-C bond oxidative aerobic cleavage.

**Scheme 17:**
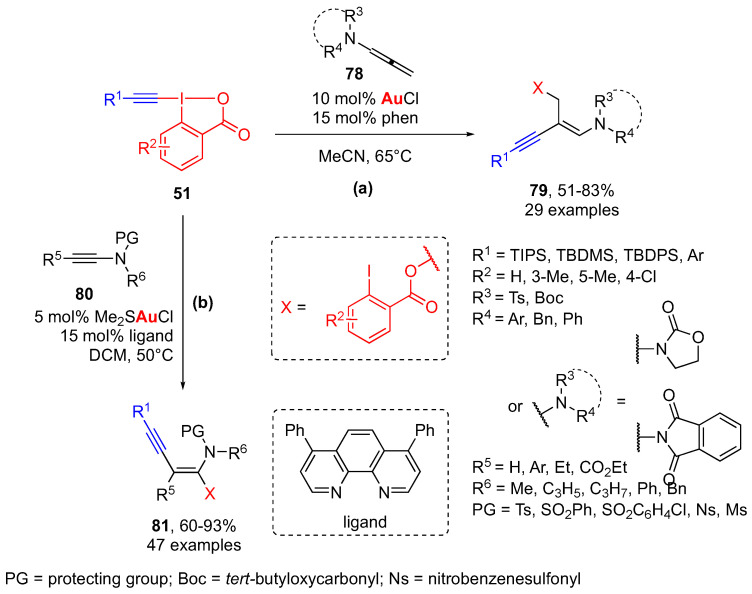
Atom economical gold-catalyzed alkynylation with the use of EBXs **51**. See explanation of reactions (**a**,**b**) in the text.

Atom economical gold-catalyzed reactions with the use of EBX were reported recently. Au-catalyzed 1,2-oxyalkynylation of *N*-allenamides **78** with ethynylbenziodoxoles **51** gives direct access to valuable 1,3-enynes **79** under mild conditions (Scheme 17a) [94].

Multisubstituted alkenes **81** are also accessible by regio- and stereo-selective gold-catalyzed acyloxyalkynylation of ynamides **80** with EBXs **51** (Scheme 17b) [95]. This efficient transformation tolerates a diverse set of functionalities, thus providing a wide range of amide enol 2-iodobenzoates **81**.

Other research groups later demonstrated that EBX reagents can be used for C−H alkynylation using a broad range of transition metal catalysts [7,9,55,58]. 

##### Copper Catalysis

**Scheme 18:**
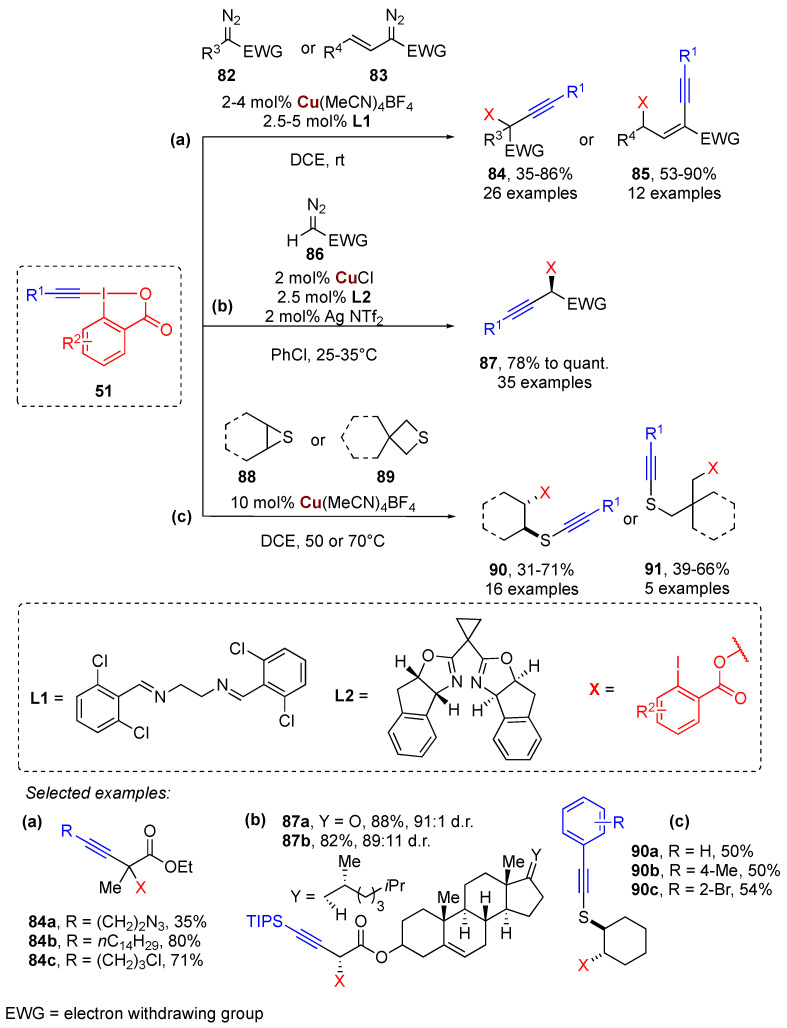
Copper-catalyzed oxyalkynylation reactions with the use of EBXs **51**. See explanation of reactions (**a**–**c**) in the text. Selected examples of products obtained in the reactions (**a**–**c**) are shown at the bottom of the scheme.

Atom economical oxyvinylation reactions of diazo compounds **82**, **83**, and **86** [96,97] and C-S bonds in thiiranes **88** and thiethanes **89** [98] using EBXs **51** under copper catalysis were reported by Waser’s group. The reaction of alkynylation of diazo compounds **82**, **83**, and **86** proceeds under mild conditions, giving highly functionalized alkynes **84**, **85**, and **87** with excellent yields and selectivities while using the inexpensive copper catalyst (Scheme 18a,b). A broad range of EBX reagents and diazo compounds were well-tolerated. Based on these investigations, the same group proposed the multicomponent copper-catalyzed reactions of diazo compounds for the synthesis of highly diverse propargylic ethers and amines [99,100].

A ring opening of thiiranes **88** and thietanes **89** using alkynylbenziodoxoles **51** and a cheap copper catalyst gives access to multifunctionalized hard-to-get thioethers **90**–**91** with moderate yields (Scheme 18c) [98].

**Scheme 19:**
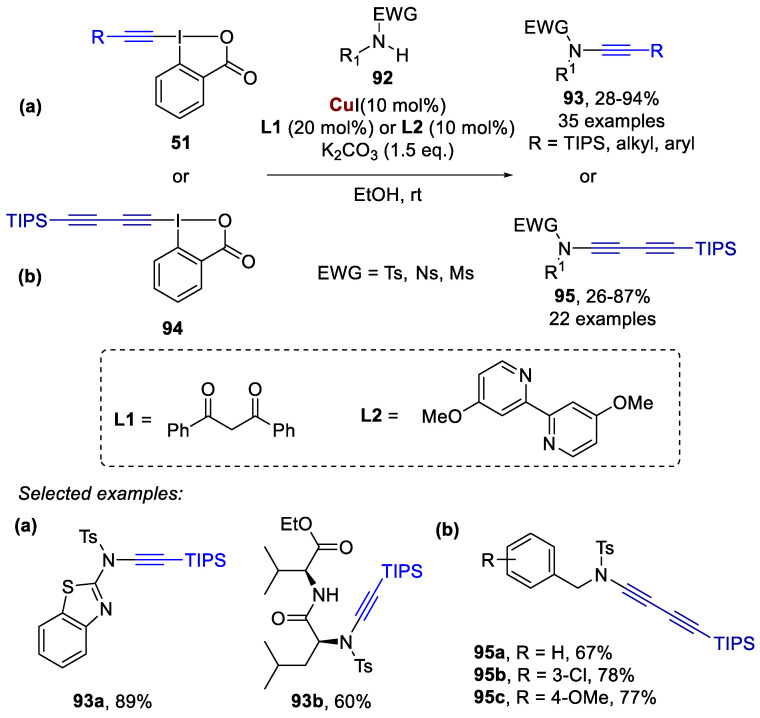
Copper-catalyzed N-alkylation reactions with the use of EBXs **51** and **94**. See explanation of reactions (**a**,**b**) in the text. Selected examples of products obtained in the reactions (**a**,**b**) are shown at the bottom of the scheme.

Fujii and Ohno reported an impressive copper-catalyzed ligand-free *N*-alkynylation of several aryltosylamides with the use of EBXs for the synthesis of the gold-catalyzed cascade cyclization substrate [101]. Inspired by the existing data, Tada and Itoh developed *N*-alkynylation of sulfonamides **92**, in which copper species allow to avoid the sterically bulky β-substituent of EBXs **51** by reacting at the α-carbon, except homo-coupling byproduct 1,3-butadiyne formation (Scheme 19a) [102]. Therefore, aryl and alkyl- sulfonamides **92,** as well as amino acids, were converted to the corresponding ynamides **93** at room temperature with broad substrate scope. The authors found that an electron-rich bidentate bipyridine ligand (4,4′-dimethoxy-2,2′-bipyridine) and protic solvent (EtOH) are critical factors to make the reaction successful, and at the time, moderately electron-poor EBXs showed higher reactivity than other EBXs. The method has been extended and applied to a late-stage diversification by copper-catalyzed azide–alkyne cycloaddition sequence in the ynamide **95** synthesis (Scheme 19b) [103]. This strategy was enabled by direct electrophilic diynylation of sulfonamides **92** with novel TIPS-diynyl benziodoxoles **94** under copper catalysis conditions.

##### Palladium Catalysis

The gold-catalyzed alkynylation of indoles **64** resulted in the C3-alkynylated products **65** (Scheme 15a) [86,104]. Under palladium catalysis, Waser and co-workers have observed very high C2 selectivity of the same reaction. Therefore, the Pd-catalyzed C2-selective direct alkynylation of 3*H*-indoles using TIPS-EBX has been developed [105]. Later, the same group proposed efficient Pd-catalyzed oxyalkylation and aminoalkylation of various alkenes with EBXs to afford heterocycles [66,106]. Multisubstituted furans can also be prepared by palladium-catalyzed condensation of *N*-aryl imines and alkynylbenziodoxoles [107,108].

**Scheme 20:**
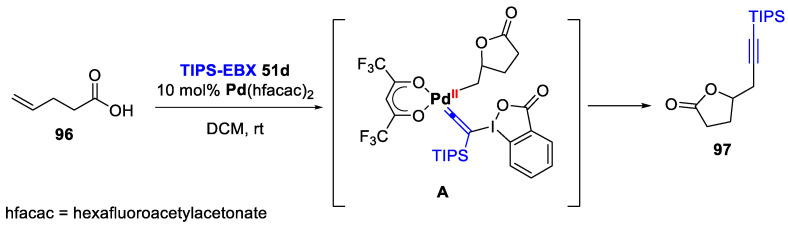
Computational mechanism of Pd(II)-catalyzed carboxyalkynylation of an olefin **96** using TIPS-EBX **51d**.

The Pd(II)-catalyzed chemical transformations using iodine(III) oxidants are most likely to proceed via a Pd(IV)/Pd(II) catalytic cycle; however, a computational mechanistic study of Pd(II)-catalyzed carboxyalkynylation of olefins **96** using TIPS-EBX **51d** has indicated that this reaction proceeds via Pd(II) vinylidene-like complex **A**, not a Pd(IV) complex, to afford product **97** (Scheme 20) [109].

**Scheme 21:**
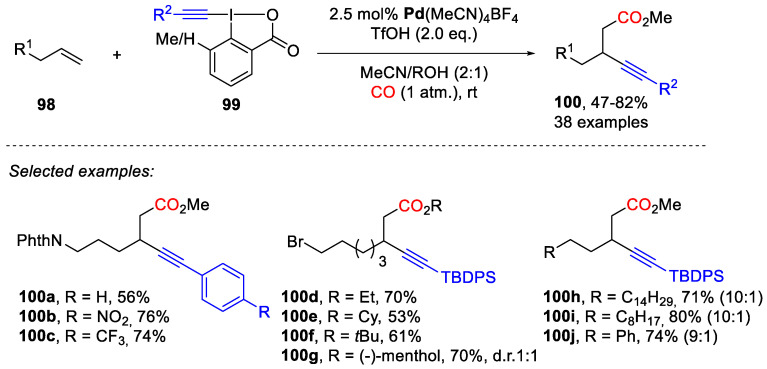
Pd-catalyzed intermolecular alkynylcarbonylation of unactivated alkenes **98** using EBXs **99**.

Convenient access to β-alkynylcarboxylic esters **100** has been achieved very recently by Pd-catalyzed intermolecular alkynylcarbonylation of unactivated alkenes **98** using EBXs **99** (Scheme 21) [110]. This method features moderate to excellent regioselectivity and excellent tolerance toward functional groups under mild reaction conditions.

Several other transition metals were investigated as catalysts in alkynylation reactions with EBXs. For example, Ru(III)- and Ir(III)-catalysts were employed for C-H alkynylation of arenes [111,112], alkenes [113], and aldehydes [114,115]; a Ag-catalyst was utilized for decarboxylative alkynylation of aliphatic carboxylic acids in aqueous solutions [116]; an Fe(III)-catalyst was applied for dehydration reaction of propargyl alcohols [117], in which TIPS-EBX serves as co-catalyst; and Pt-catalyzed domino cyclization-alkynylations were developed as well [118,119,120].

#### 3.2.2. Photocatalysis

**Scheme 22:**
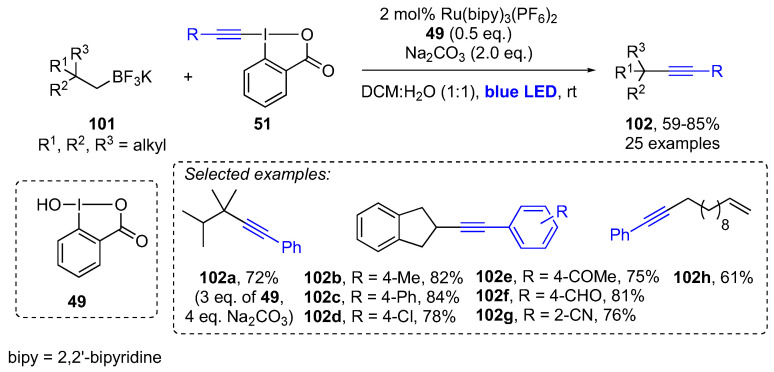
Deboronative alkynylation with the use of EBXs **51** under photoredox catalysis.

Ethynylbenziodoxoles were also used for radical alkynylation under photoredox reaction conditions [9,56]. A visible light-induced chemoselective deboronative alkynylation of primary, secondary, and tertiary alkyl trifluoroborates or boronic acids **101** with the use of alkynylbenziodoxoles **51** have been developed in 2014 by Chen’s group (Scheme 22) [71]. This reaction is highly chemoselective and performs well on substrates containing alkenes, alkynes, aldehydes, ketones, esters, nitriles, azides, aryl halides, alkyl halides, alcohols, and indoles, with no detectable occurrence of side reactions, and can be carried out in neutral aqueous conditions to generate aryl, alkyl, and silyl substituted alkynes **102**. Later, Chen and co-workers studied the radical acceptor and oxidative quencher reactivity of EBXs, in which unsubstituted EBXs played balancing roles in both processes, while electron-rich benziodoxole derivatives demonstrate synthetic advantages in some cases [121]. Very recently, Waser’s group proposed a one-pot, two processes for EBX generation and their direct application in substrate functionalization, such as deboronative alkynylation as well as thioalkynylation, O-VBX formation, β-ketoester alkynylation, oxy-alkynylation, decarboxylative alkynylation, and double thiol addition [82].

**Figure 2:**
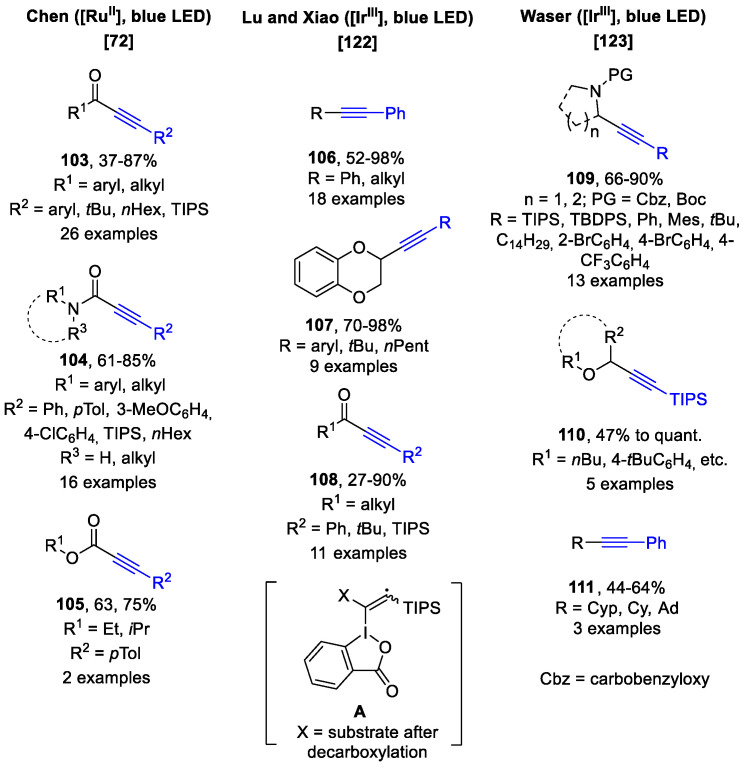
Products **103**–**111** and intermediate **A** of Ru(II) or Ir(III) photoredox catalyzed alkynylation with the use of EBXs [72,122,123].

In 2015, three research groups proposed decarboxylative alkynylation of various carboxylic acids using Ru(II) or Ir(III) photoredox catalysis and EBXs independently [72,122,123]. A plethora of ynones **103**, **108**, ynamides **104**, **109**, ynoates **105**, **110,** and alkynes **106**, **107**, and **111** can be prepared under mild reaction conditions (Figure 2). The reaction proceeds via intermediate **A**, which is a result of the α-addition of substrate radical to EBXs. Then, intermediate **A** undergoes a subsequent radical elimination to yield a desired product and benziodoxolyl radical. Aldehydes can also be converted into various ynones, ynamides, and ynoates using EBXs under Ir(III) photoredox catalysis [124] and under transition metal-free conditions, with the use of *tert*-butyl hydroperoxide as radical initiators and EBXs [125].

At the same time, Wang and co-workers have developed similar radical alkynylation of α-keto acids with bromoacetylenes catalyzed by IBA **49** [126]. The reaction proceeds under sunlight irradiation without the use of photo- or metal-catalysts. The authors proposed in situ formation of EBX during the reaction. This alkynylation tolerates a series of substituted groups and affords ynones in good yields. In parallel, Duan et al. utilized the EBXs-K_2_S_2_O_8_ system for decarboxylative alkynylation of α-keto acids and oxamic acids in aqueous media to afford similar ynones with moderate to high yields [127]. Another transition metal-free synthesis of ynones from aldehydes has been performed with the use of EBX reagents and an excess of radical initiators (*tert*-butylhydroperoxide) at 100 °C in DCE [128].

**Scheme 23:**
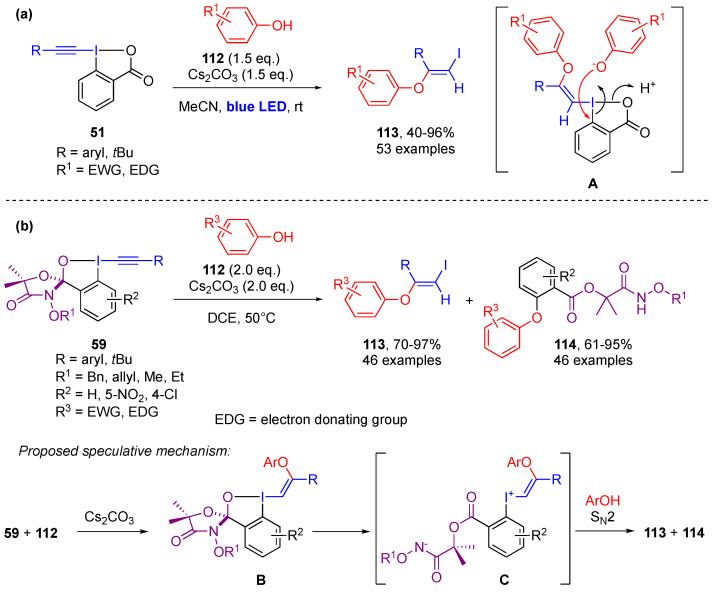
Preparation of (*Z*)-2-iodovinyl phenyl ether derivatives **113** and diaryl ethers **114** with the use of EBX **51** and **59,** and phenols **112**. See explanation of reactions (**a**,**b**) in the text.

Miyake et al. reported light-driven intermolecular charge transfer-induced reactivity of EBXs **51** and phenols **112** to afford a diverse array of (*Z*)-2-iodovinyl phenyl ether derivatives **113,** with excellent regio- and stereo-selectivity under irradiation with visible light (Scheme 23a) [129]. The authors assumed a photoinduced electron transfer step involving an intermediary vinylbenziodoxolone–phenoxide EDA complex **A** that subsequently leads to unprecedented phenyl−I bond cleavage.

Very recently, a similar base-promoted metal-, photocatalyst- and light-free reaction of phenols **112** with spirocyclic EBXs **59** has been developed to construct both the (*Z*)-2-iodovinyl aryl ethers **113** and diaryl ethers **114** (Scheme 23b) [130]. To generate the two desired products, the authors proposed a S_NAr_2 reaction of phenol **112** with vinyl aryl iodonium salts intermediates **C** that subsequently leads to the phenyl-I bond cleavage and phenyl-O bond formation. The authors assumed that the vinyl aryl iodonium salts **B** was formed in situ from the spiro-*cis*-β-phenol-VBXs **B**, which was generated between electrophilic spiro-EBXs **59** and nucleophilic arylols **112**.

**Scheme 24:**
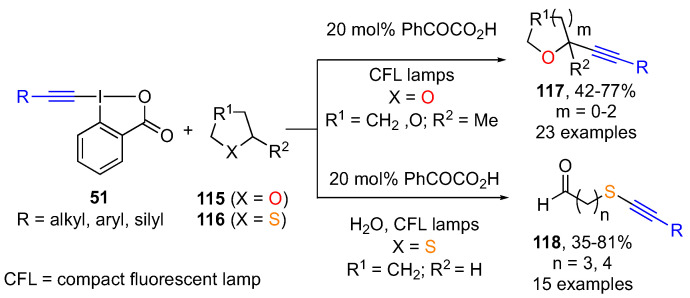
Photocatalytic direct C-H alkynylation of ethers **115** and the deconstructive alkynylation of thioethers **116** using alkynylbenziodoxoles **51**.

A combination of non-metallic photocatalysts/EBXs has been employed effectively in the alkynylation reaction. An efficient method for the direct C-H alkynylation of ethers **115** and the deconstructive alkynylation of thioethers **116** using alkynylbenziodoxoles **51** have been developed recently (Scheme 24) [131]. This photochemical alkynylation was performed utilizing phenylglyoxylic acid as the photoinitiator under household fluorescent light bulb irradiation. Cyclic ethers **115** have been alkynylated at the α-position to afford products **117**; meanwhile, the oxidative ring-opening reaction of S-heterocycles **116** led to thioalkynylated aldehydes **118**. The latter transformation is unprecedented and proceeded in high yields with only a few sulfur oxidation side products. Non-cyclic thioethers were alkynylated as well with moderate to good yields, while protected carbohydrates and amino acids gave lower yields of products. The authors also mentioned that the alkynylation of N-heterocycles was not successful.

**Scheme 25:**
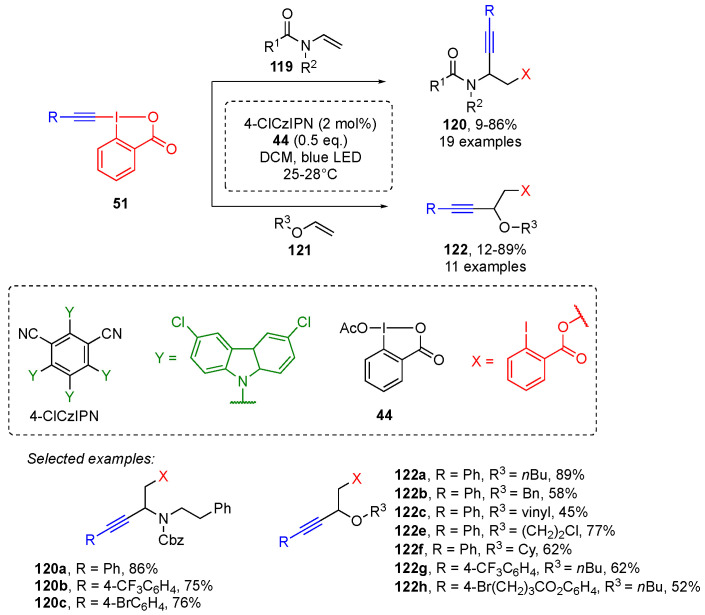
Organophotocatalytic atom economical 1,2-oxyalkynylation of ene-carbamates **119** and enol ethers **121** using EBXs **51** and hypervalent iodine catalyst **44**.

Organophotocatalytic atom economical 1,2-oxyalkynylation of ene-carbamates **119** and enol ethers **121** using EBXs **51** in presence of hypervalent iodine compound **44** at room temperature affords functionalized amides **120** and ethers **122** in high yields (Scheme 25) [132]. An ene-carbamate radical cation is a key intermediate that ensures the *anti*-Markovnikov regioselectivity initiated by nucleophile addition, contrasting with the classical atom transfer radical addition mechanism usually invoked for the functionalization of alkenes with hypervalent iodine reagents [132].

**Scheme 26:**
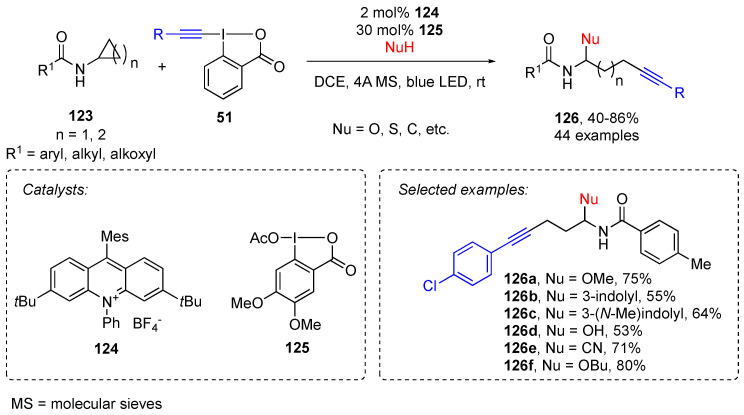
Selective C(sp^3^)-C(sp^3^) cleavage/alkynylation of cycloalkylamides **123** using EBXs **51** and **125** as co-catalysts.

Aminoalkynes **126** with versatile alkyne and amine substituents are efficiently constructed from cycloalkylamides **123** via amidyl radicals enabled by EBXs **51** (Scheme 26) [133]. A catalytic amount of cyclic iodine(III) **125** facilitated the single-electron oxidation and ring-opening alkynylation of cycloalkylamides **123**. The authors assumed a noncovalent activation of hypervalent iodine(III) reagents on the cycloalkylamides, which shows a vast difference compared to the covalent carboxylate/alcohol activation. Various α-amino-substitutions on the aminoalkynes **126** can be easily introduced by oxygen, sulfur, and carbon nucleophilic trapping, and the aminoalkyne products can readily derivatize to various fused azacycles with bioactivities.

The radical reaction of SF_5_Cl with ethynylbenziodoxoles under blue LED irradiation gave the desired SF_5_-substituted alkynes in moderate to high yields [134]. The pentafluorosulfanyl derivatives have potential applications in materials and drug design, and served as valuable synthetic building blocks [135,136,137,138,139].

#### 3.2.3. Transition Metal-Free Reactions

**Scheme 27:**
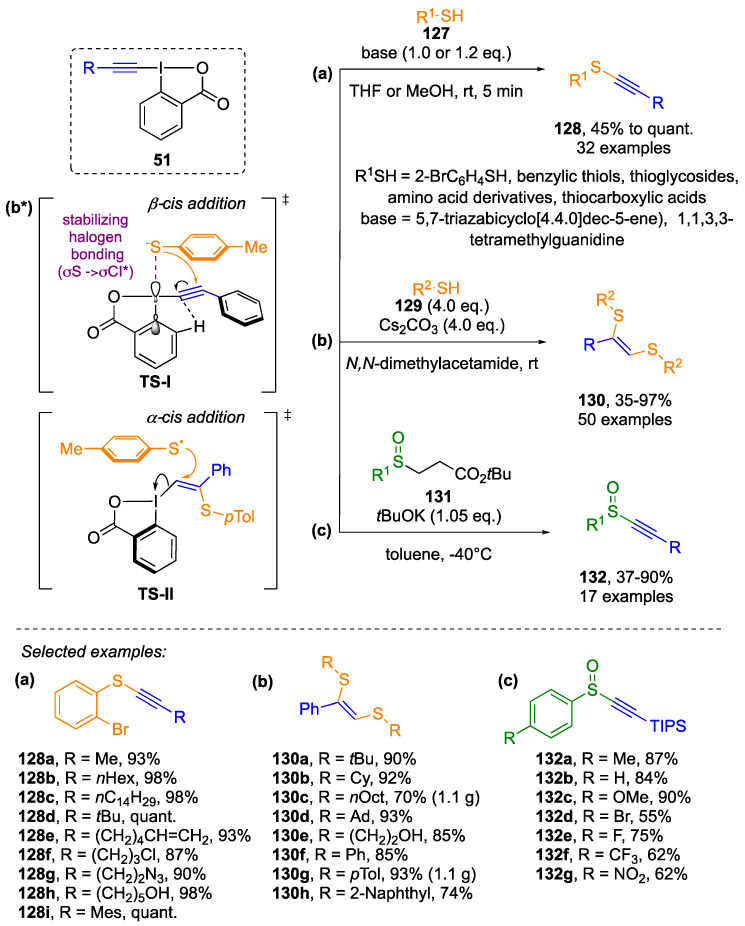
Alkynylation reactions of thiols **127**, **129** and sulfenates **131** using EBXs **51**. See explanation of reactions (**a**–**c**) in the text. Selected examples of products obtained in the reactions (**a**–**c**) are shown at the bottom of the scheme. Structures of the transition states in the mechanism of reaction (**b**) are shown under (b*).

Functionalized alkyl- and aryl-substituted EBX reagents **51** have been used for the alkynylation of both aromatic and aliphatic thiols **127** to afford thioalkynes **128** in moderate to quantitative yields (Scheme 27a) [140]. Functional groups such as alkenes, alkynes, ethers, chlorides, azides, and alcohols were tolerated on the alkynes. In addition to simple thiophenols and benzylic thiols, the alkynylation of cysteine in a dipeptide, thioglycosides, thiobenzoic acid derivatives, and sodium hydrogen sulfide was also successful. Later, the alkynylation method has been efficiently used in the functionalization of cysteine residues in complex proteomes due to user-friendly aspects of the method, such as a 5 min reaction time, open-flask, water tolerance, and ambient temperature [141,142,143]. The method has been recently applied as the second step in a one-pot thioalkylation reaction [82].

A general and efficient strategy for the synthesis of 1,2-dithio-1-alkenes **130** with excellent regioselectivity and stereoselectivity has been presented through unprecedented reactivity between the EBXs **51** and the thiols **129** (Scheme 27b) [144]. This operationally simple procedure utilizes mild conditions, resulting in a broad substrate scope and high functional group tolerance. The *cis* regioselectivity observed in the final products is created through a combination of two steps: *cis*-selective nucleophilic R^1^SH addition (**TS-I**) followed by a *cis*-specific radical R^1^SH addition (**TS-II**) (Scheme 27b*). Interestingly, different inorganic salts accelerate the reaction by acting as basic additives in the first RSH addition. Under the standard reaction conditions using Cs_2_CO_3_, the results suggest that the rate-limiting step is the formation of R^1^S• radicals from R^1^SH that takes place before the second R^1^SH addition. In addition, the method was effectively applied to the synthesis of a few examples of benzo-1,4-dithiines.

Alkynyl sulfoxides **132** can be efficiently synthesized under transition metal-free conditions from corresponding sulfenates **131** and EBXs **51** through retro Michael elimination initiated by *tert*-butoxide at low temperature and subsequent unstable sulfenate anion addition (Scheme 27c) [78]. The trapping of the resulting sulfenate anions with EBX reagents afforded alkyl and aryl alkynyl sulfoxides **132** in high yields. Additionally, two aryl vinyl sulfoxides were also isolated when using VBX reagents.

**Scheme 28:**
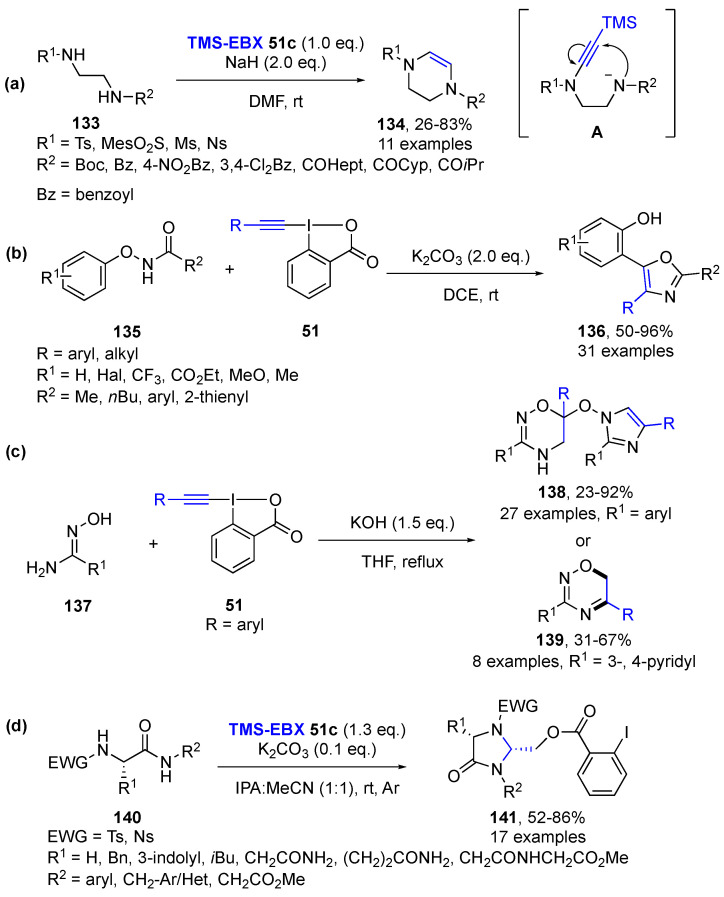
Transition metal-free EBXs **51**-mediated synthesis of heterocycles. See explanation of reactions (**a**–**d**) in the text.

A wide range of heterocycles can be synthesized using EBXs under transition metal-free conditions [70,145,146,147]. Cossy et al. reported the synthesis of tetrahydropyrazines **134** from diamides **133** using TMS-EBX **51c** in the presence of a strong base through 6-*endo-dig* cyclization of the ynamide intermediate **A** (Scheme 28a) [70]. A mild and straightforward synthetic protocol for the construction of 2-(oxazol-5-yl)phenol derivatives **136** promoted by K_2_CO_3_ from *N*-phenoxyamides **135** and alkynylbenziodoxoles **51** at room temperature has been developed through sequential [3,3]-rearrangement/alkylidene carbene insertion/Michael addition/cyclization (Scheme 28b) [70]. Later, the same group proposed transition metal-free substituent-controlled synthesis of two kinds of functionalized oxadiazine derivatives **138–139** from EBXs **51** and amidoximes **137** under one-base conditions (Scheme 28c) [146]. This strategy is very challenging because EBXs could form an O-vinylbenziodoxole intermediate, which can undergo two different 1,2-migration processes leading to two different oxadiazine derivatives. Another striking feature of the reaction is the switchable selectivity of EBXs to synthesize oxadiazine derivatives by adjusting substituent R^1^ of the amidoximes **137**.

Very recently an atom economical synthesis of 4-imidazolidinones **141** from diamides **140** and TMS-EBX **51c** via unprecedented double Michael-type addition under basic conditions has been proposed (Scheme 28d) [147].

**Scheme 29:**
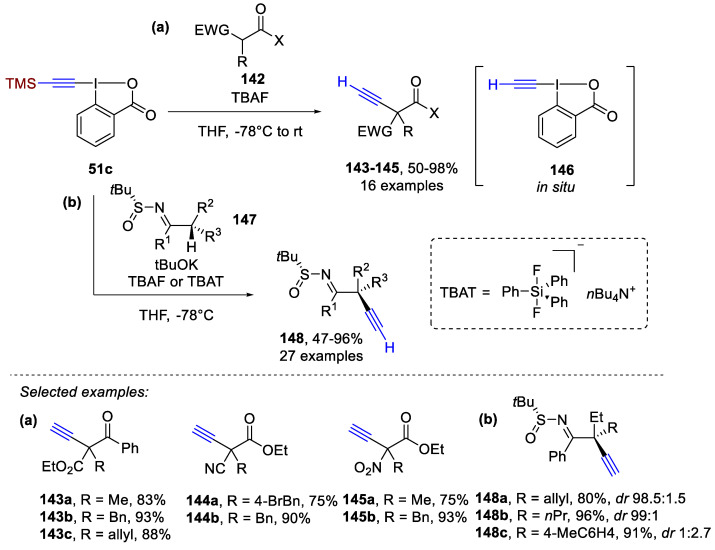
Alkynylation of activated carbonyl compounds **142** and α,α-disubstituted *N*-*tert*-butanesulfinyl ketimines **147** with the use of alkynylbenziodoxole **51c**. See explanation of reactions (**a**,**b**) in the text. Selected examples of products obtained in the reactions (**a**,**b**) are shown at the bottom of the scheme.

Numerous works were dedicated to the alkynylation of activated carbonyl compounds with the use of EBXs [67,68,148,149,150]. In the pioneering work [67], Waser et al. proposed the ethynylation of keto, cyano, and nitroesters **142** with H-EBX **146**, which is generated in situ from alkynylbenziodoxole **51c** by TBAF treatment at low temperature (Scheme 29a). In their next work [68], an alkynylation method of cyclic keto esters was improved, as well as the scope of starting EBX reagents. Further reports concern the variations of reaction conditions (changing the base, additives, and temperature) and either carbonyl compounds or EBXs and, consequently, the scope of obtained products [148,149,150].

Stereoselective electrophilic α-alkynylation of α,α-disubstituted *N*-*tert*-butanesulfinyl ketimines **147** using TMS-EBX **51c** in the presence of fluoride have been proposed (Scheme 29b) [151]. Despite the steric and electronic similarity between the two α-substituents, the entire reaction proceeded in a strongly stereoselective manner: *t*BuOK promoted α-deprotonation of the acyclic ketamine **147** to generate stereodefined fully substituted aza-enolates, which stereoselectively formed C−C bonds with electrophilic alkynylation reagents, affording α-alkynylation products **148** with excellent stereocontrol.

## 4. Vinylbenziodoxoles (VBXs)

### 4.1. Synthesis and Structure

The interest in vinylbenziodoxoles (VBXs), also named alkenylbenziodoxoles, has recently significantly increased. Earlier works described the formation of VBXs as products in various addition reactions of alkynylbenziodoxoles [140,152,153]; however, the reactivity of VBXs was systematically investigated only in the last five to six years. In a recent review [10], various approaches to the synthesis of VBX reagents and their reactivity were described in detail; therefore, below we will consider only the main aspects and recent findings.

#### 4.1.1. C-VBXs

**Scheme 30:**
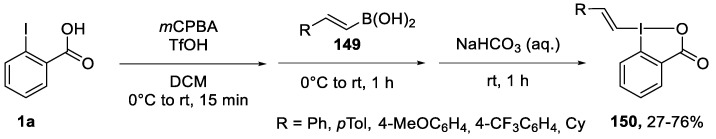
One-pot synthesis of C-VBX **171**.

In general, the vinylbenziodoxoles can be further classified as X-VBX and C-VBX, containing either heteroatom X or carbon substituent at the β-carbon of the vinyl moiety, respectively. Several examples of the preparation of C-VBX by a coupling reaction of various vinylboronic acids and hypervalent iodine compounds have been reported [154,155,156,157]. In 2016, Olofsson and co-workers proposed a one-pot synthesis of C-VBX **150** starting from 2-iodobenzoic acid **1a** (Scheme 30) [154].

**Figure 3:**
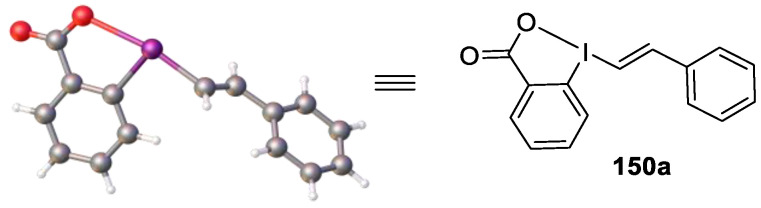
X-ray structure of vinylbenziodoxole **150a** [154].

The cyclic structure of VBXs was confirmed by X-ray analysis of the styrylbenziodoxole **150a** (Figure 3) [154]. The molecular structure has a distorted T-shape with an O-I-C(sp^2^) angle of 165.88 Å, which is similar to the reported arylbenziodoxoles [23,29,30,31] and alkynylbenziodoxolones [61,62,69]. The endocyclic I-O bond length of 2.51 Å is significantly longer than in alkynylbenziodoxoles [61,62,69], and in general, it is close to the I-O bond in the structure of arylbenziodoxolones [23,29,30,31]. This bond length trend is in agreement with the larger trans influence exerted by vinyl and aryl groups compared to alkynyl- and trifluoromethyl groups. The trans influence correlates with the Hammett inductive constants, which are similar for vinyl and phenyl groups [158,159].

Despite the good tolerance of the functional groups in the alkene moiety and aromatic core, the main limitations of the synthesis of C-VBXs still need to be highlighted. Firstly, alkenyl boronic acids as a vinyl source are difficult to handle, and the use of other alkenyl- precursors was not successful so far. Secondly, the existing methods can be used only for the preparation of (*E*)-C-VBXs, and the synthesis of (*Z*)-isomers remains unknown, probably due to decomposition and isomerization issues [160,161].

**Scheme 31:**
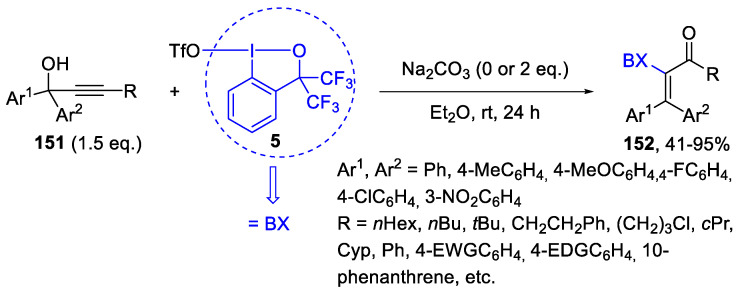
Synthesis of vinylbenziodoxoles **152** from the benziodoxole triflate **5** and propargylic alcohols **151**.

Another interesting and facile preparation of C-VBXs was proposed by Yoshikai and co-workers [162]. Benziodoxole triflate **5**, acting as an electrophile, promotes an iodo(III)-Meyer–Schuster rearrangement of propargylic alcohols **151** under simple and mild conditions to give α-λ^3^-iodanylenones **152** in moderate to good yields (Scheme 31). This transformation tolerates a wide range of functionalized propargylic alcohols **151**, thus complementing the previously reported halogen-intercepted Meyer–Schuster rearrangement [163,164,165,166]. The α-λ^3^-iodanylenones **152** can be used for Pd-catalyzed cross-coupling reactions to afford multisubstituted enones.

#### 4.1.2. X-VBXs

**Scheme 32:**
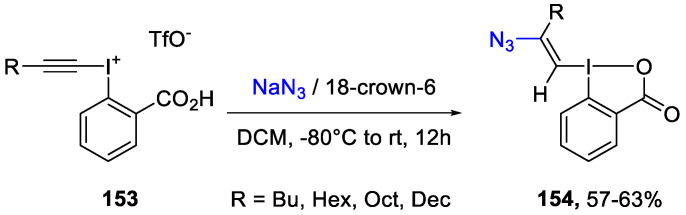
First reported synthesis of vinylbenziodoxoles **154**.

In contrast to C-VBX, numerous papers reporting the addition reaction of S-, N-, O-, and X- nucleophiles to ethynylbenziodoxoles (EBXs) leading to the formation of various X-VBXs were published in the last 5 years [162,167,168,169,170,171,172,173,174,175]. In their pioneering publication, Kitamura and co-workers reported that the additional reaction of azide anion to alkynyl(*o*-carboxyphenyl)iodonium triflate **153** affords vinyl-substituted cyclic iodanes **154** as trans isomers (Scheme 32), which is in contrast to the previously known reactions of the non-cyclic alkynyliodonium salts [152].

- Common approach starting from EBXs

The preparation of VBXs by addition reactions of various nucleophiles with EBXs **51** or **54** under different conditions is summarized in Table 1.

Waser’s group investigated reactions of S-nucleophiles **155** and revealed some mechanistic insights into the formation of X-VBX **162** (Table 1, entry 1) [140]. Furthermore, they successfully applied hypervalent iodine chemistry for the fast and selective peptide and protein **156** modification to obtain **163** (Table 1, entry 2) [140,141,142,143]. Various *cis*-β-*N*-derivatives of VBX **164–166** can be prepared from the EBX **51** with the use of catalytic amounts of the base at ambient temperature (Table 1, entries 3–5) [167,169]. The synthesis of O-VBXs **170** is also possible under basic conditions from phenols **160** and EBXs **51,** as it is for N- and S-VBXs (Table 1, entry 9).

**Scheme 33:**
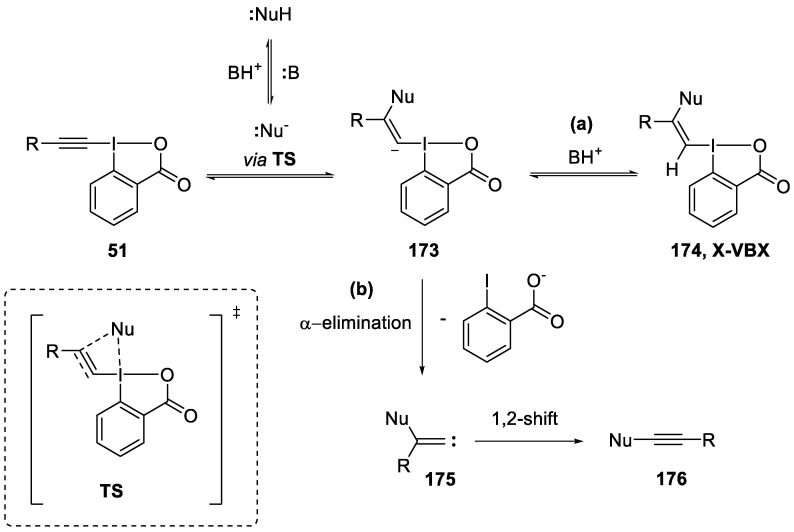
Proposed general mechanism of stereoselective formation of X-VBXs **174** (**a**) and competitive alkynylation process (**b**).

According to the data on the mentioned transformations (Table 1, entries 1–5, 9) and the detailed mechanistic investigation of the alkynylation of thiols [177], we can summarize the mechanism of stereoselective formation of X-VBXs **174** in the presence of a base (Scheme 33). Initially, the nucleophile adds to the β-carbon of EBX in the presence of a base to form vinyl anion **173** via preliminary coordination of nucleophile to the iodine center **TS**. Subsequently, **173** is effectively protonated to afford X-VBX **174**. The equilibrium between **51**, **173,** and **174** lies strongly in favor of **174,** allowing its isolation once the reaction mixture is neutralized. On the other hand, **173** reacts slowly and irreversibly via carbene **175** to form alkyne **176**. Higher base concentration leads to an increased amount of anion **173**, resulting finally in full conversion to alkyne **176**.

Yoshikai and co-workers reported unprecedented Pd-catalyzed stereoselective 1,2-iodine(III) shift/1,1-difunctionalization affording (*E*)-O-VBXs **168–169** (Table 1, entries 6, 8). The reaction involves a Pd-assisted 1,2-iodine(III) shift of the EBX followed by a stereoselective introduction of functionality into the α-position of the transient Pd-vinylidene species **B** to give intermediate **C** (Table 1, entry 7) [153,176]. The products **168** and **169** of this 1,1-difunctionalization reaction serve as new building blocks for further synthetic transformations; for instance, Stille coupling and Sonogashira coupling [153]. Interestingly, Heck reaction conditions led to the decomposition of O-VBX **168–169**, whereas a simple exposure of the latter to methyl acrylate resulted in clean *E/Z* isomeriozation to afford the *Z*-isomer [153].

In 2019, Yoshikai et al. reported *anti*-hydrochlorination and *syn*-iodochlorination of EBXs **51** using pyridine hydrochloride as an HCl source and iodine monochloride, respectively, for the synthesis of highly functionalized Cl-VBXs **171–172** (Table 1, entry 10) [170]. It should be noted that a narrower scope was observed for the iodochlorination, and alkyl-EBXs were not tolerated in the transformation. Nevertheless, both reactions were achieved using extremely simple reagents under mild, open-air conditions with high stereoselectivity.

- One-pot approach starting from other iodine (III) reagents

**Scheme 34:**
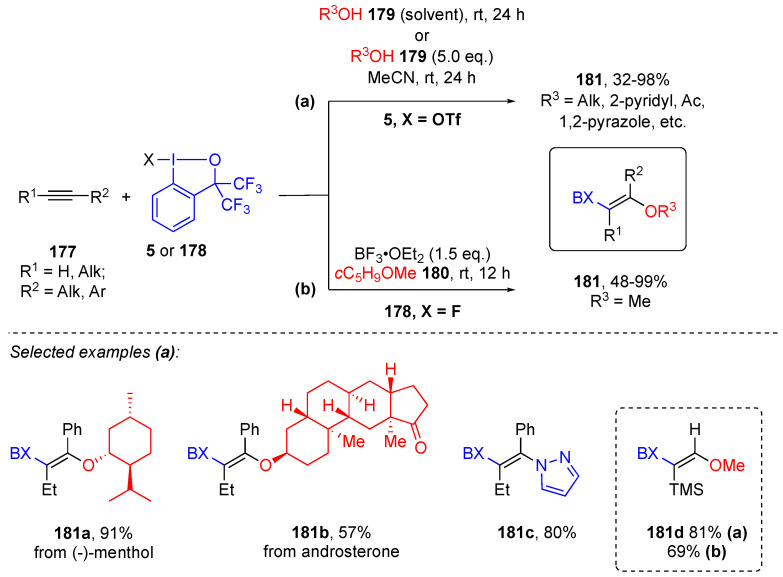
Synthesis of highly substituted X-VBXs **181** from iodine(III) reagents **5** (**a**) and **178** (**b**).

Several recent works by Yoshikai’s group were dedicated to the synthesis of highly substituted X-VBXs [162,171,172,173,174,175]. Among them is a stereoselective synthesis of vinyl ethers **181** via *trans*-difunctionalization of terminal and internal alkynes **177** by alcohols **179** or cyclopenthyl methyl ether (CPME) **180** and iodine(III) reagents **5** or **178** (Scheme 34a,b respectively) [171,172].

In contrast to general outcomes, trimethylsilylacetylene underwent the iodo(III)-etherification with opposite regioselectivity to afford the β-silyl vinyl ether **181d** in 81% yield, presumably due to the ability of the silyl group to stabilize positive charge at the β-position [178]. Except for **181d**, the **178**/BF_3_/CPME system [171] gave better yields or proved equally efficient compared with the **5**/MeOH system [162]. Both approaches have high tolerance toward a variety of functionalized internal and terminal alkynes **177,** as well as various alcohols **179** (Scheme 34a), affording β-λ^3^-iodanyl vinyl ethers in good yields with high regio- and stereo-selectivities. The benziodoxole moiety (BX) of the products can be used as versatile precursors for the synthesis of structurally diverse stereochemically well-defined vinyl ethers that are difficult to access by other methods.

**Scheme 35:**
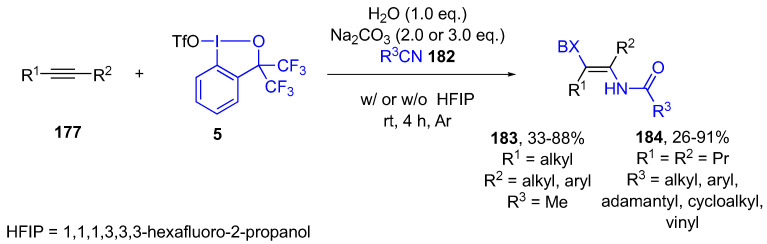
Stereoselective synthesis of highly substituted N-VBXs **183**–**184** from iodine(III) reagent **5**, nitriles **182,** and alkynes **177**.

In continuation of these works, a Ritter-type *trans*-difuctionalization of alkynes **177** mediated by the trivalent iodine electrophile **5** for the stereoselective synthesis of multisubstituted enamides **183–184** has been developed (Scheme 35) [173]. The reaction conditions were carefully investigated including the water content and the reaction medium, and a variety of internal alkynes **177,** as well as nitriles **182,** were found to be applicable for the reaction to afford *trans*-iodanyl enamides **183–184** in moderate to good yields. Transformations of the C-I(III) bond and subsequent synthetic applications were demonstrated.

**Scheme 36:**
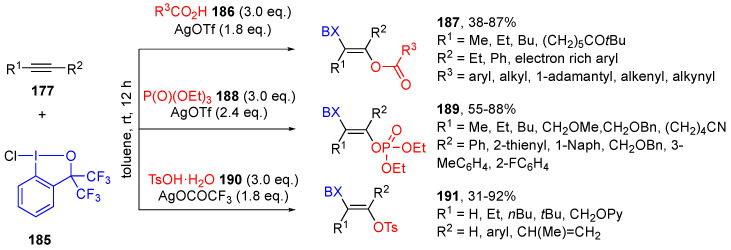
Esterification-iodo(III)-functionalization of alkynes **177**.

Very recently, the synthesis of β-iodo(III)enol carboxylates **187**, phosphates **189**, and tosylates **191** through regio- and stereo-selective iodo(III)functionalization of alkynes **177** was reported (Scheme 36) [175]. The combination of chlorobenziodoxole **185** and silver salt generates a cationic iodine(III) electrophile to activate alkynes **177** and involve different carboxylic acids **186**, triethyl phosphate **188,** and *p*-toluenesulfonic acid **190** as nucleophiles in an addition reaction.

**Scheme 37:**
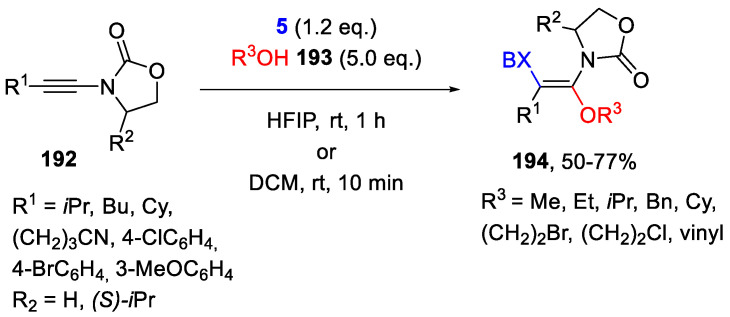
A *trans*-iodo(III)etherification reaction of ynamides **192** with benziodoxole triflate **5** and alcohols **193**.

The approach to the synthesis of O-VBXs [172] was modified and applied for the preparation of β-alkoxy-β-amido vinylbenziodoxoles **194** via *trans*-iodo(III)etherification reaction of ynamides **192** with benziodoxole triflate **5** and alcohols **193** (Scheme 37) [174]. Despite the intrinsic susceptibility of electron-rich ynamides and enamides toward Brønsted acid, the desired β-alkoxy-β-amido VBXs **194** could be obtained in moderate to good yields under carefully controlled reaction conditions. High *trans*-selectivity of the reaction, as well as the ability to install a chiral oxazolidinone moiety, allows the use of β-alkoxy-β-amido VBXs in stereoselective transformations.

**Scheme 38:**
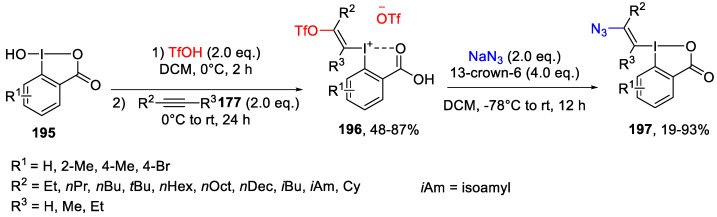
Synthesis of β-azido vinylbenziodoxoles **197**.

Finally, the reaction of vinylic nucleophilic substitution of pseudocyclic β-trifluorosulfonyloxy vinylbenziodoxoles **196** with azide anion allows obtaining β-azido vinylbenziodoxoles **197** with the retained configuration of double bond (Scheme 38) [179]. Starting pseudocyclic β-trifluorosulfonyloxy vinylbenziodoxoles **196** can be easily prepared using a one-pot procedure from hydroxybenziodoxoles **195** using TfOH [179] or Tf_2_O [180] treatment and an additional reaction of alkynes **177**.

### 4.2. Synthetic Applications

VBXs are used as synthetic equivalents of the alkenyl group. Only several publications are dedicated to the systematic studies of the reactivity of VBXs in vinylation reactions. The vast majority of works report isolated examples of VBX reactivity as part of broader studies in the field of hypervalent iodine chemistry; some of these works were summarized in a recent review [10]. In this section, only systematic studies of VBXs, as well as new examples of specific applications, will be considered.

#### 4.2.1. Metal-Free Reactions

**Scheme 39:**
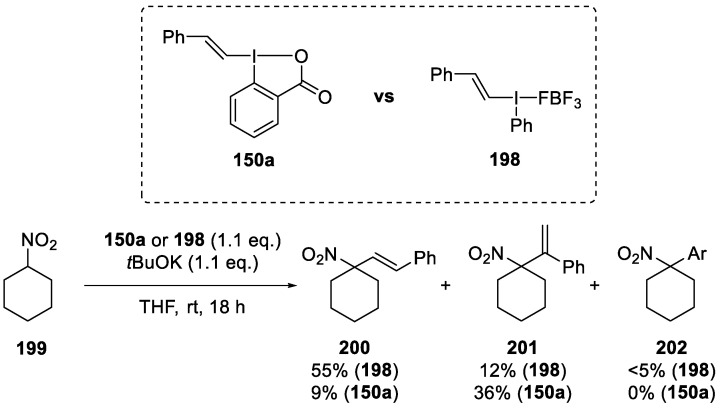
Reactivity difference between C-VBX **150a** and acyclic vinyliodonium salt **198**.

The first study of the vinylation reaction of C-VBX was published by Ochiai and coworkers in 1997. It was demonstrated that the reactions between C-VBX **150a** and nitrocyclohexane **199** resulted in the regioselective formation of terminal alkene **201**, which was opposite to the regioselectivity observed with acyclic vinyliodonium salt **198** [181] when the major product is internal alkene **200** (Scheme 39) [154]. In the reaction of C-VBX **150a**, product **201** was isolated as a major product in 57% yield, when the reaction was performed with 2.0 eq. of **150a** in DME for 72 h. The unusual product distribution indicates a different mechanistic pathway not involving radical intermediates [154].

**Scheme 40:**
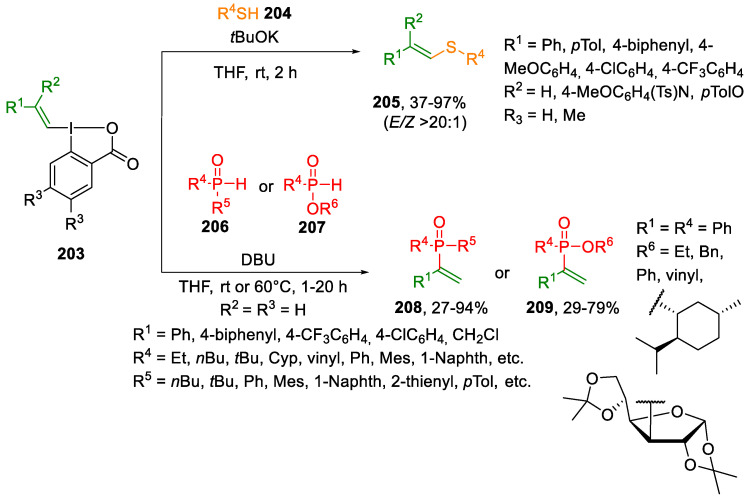
C-VBX **203** mediated vinylation reactions of thiols **204**, phosphine oxides **206,** or H-phosphinates **207**.

The group of Olofsson employed C-VBXs **203** to vinylate a range of aliphatic and aromatic thiols **204** [157] and phosphine oxides **206** or H-phosphinates **207** [182] under mild and transition metal-free conditions (Scheme 40). The reported approaches allow for *E*-alkenyl sulfides **205** to synthesize, as well as terminal alk-1-enyl phosphine oxides **208** and alk-1-enyl phosphinates **209** with complete chemo- and regio-selectivity and good yields.

**Scheme 41:**
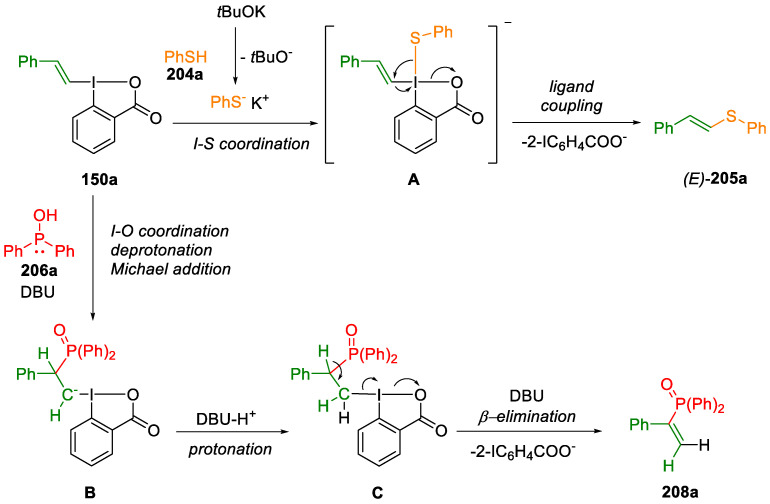
Proposed mechanistic pathways of P- and S-vinylation with the use of C-VBX **150a**. Structures of key intermediates **A**–**C** are shown.

Later Olofsson et al. conducted a detailed mechanistic study of C-VBX vinylations, including NMR studies, deuterium labeling, and computations, to figure out the observed regio- and stereo-chemical outcome (Scheme 41) [183]. According to this study, C-VBXs react by two different pathways leading either to the internal (**205,** Scheme 40) or the terminal (**208** and **209,** Scheme 40) alkene. Deuterium-labeling studies and computations support that the S-vinylation of thiol **204a** proceeds through deprotonation followed by a ligand coupling to provide intermediate **A** and then the final internal alkenes **205a** with retained *E*-configuration. The P-vinylation of diarylphosphine oxides **206a** instead begins with I−O coordination of the corresponding phosphinous acid to C-VBX **150a**, then simultaneous deprotonation and Michael-type addition leading to anionic intermediate **B**, which then transforms to the terminal alkene **208a** through a base-assisted protonation (intermediate **C**) and E2 elimination. In this work, the general regioselectivity trend for VBX vinylations under metal-free conditions was predicted, where ambident nucleophiles will deliver terminal alkenes, whereas monodentate or strong nucleophiles will provide internal alkenes.

**Scheme 42:**
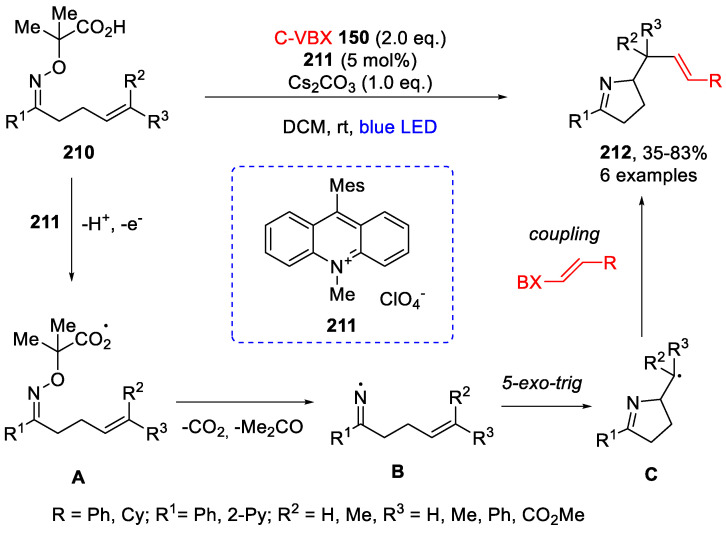
Free-radical photoredox vinylation of oximes **210** using C-VBX **150**. Structures of key intermediates **A**–**C** are shown.

In 2017, Leonori’s group reported the first use of C-VBX **150** as coupling partners in a free-radical photoredox process in the presence of acridinium dye **211** (Scheme 42) [184]. A nitrogen-centered radical **B** was generated through a photoredox-initiated decarboxylation of oxime **210** via carboxy radical **A**, followed by cyclization to give alkyl radical **C**, which was trapped to afford nitrogen heterocycles **212**. In the next step, C-VBX **150** was effectively employed as a radical trap with the complete retention of the alkene (*E/Z*) ratio to give products **212**. Cy-VBX was also used in a single example to give the final product **212** (R^1^ = Ph, R^2^ = R^3^ = Me) in a 35% yield.

**Scheme 43:**
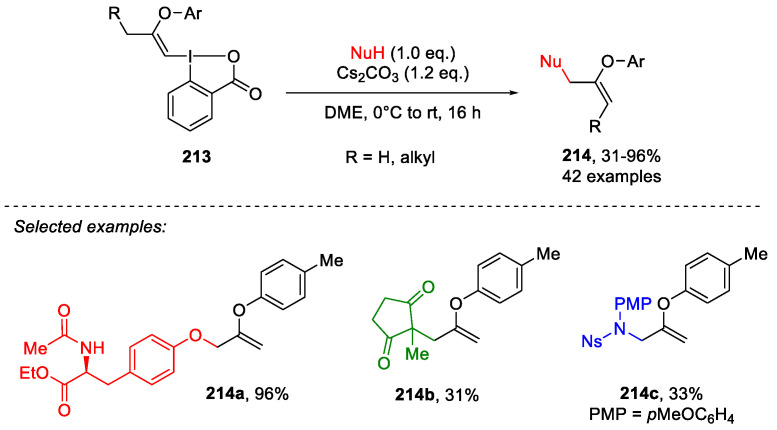
Vinylation of O-, N-, and C-nucleophiles using O-VBXs **213**.

An umpolung strategy of enol ethers to generate oxy-allyl cation equivalents from O-VBXs **213** under mild basic conditions was reported [185]. A plethora of vinylated compounds **214** were obtained stereoselectively using O-, N-, and C- nucleophiles, including natural products and O-VBXs **213** (Scheme 43). The reaction was most efficient for phenols as nucleophiles, but the conditions were applied to the reaction with C- and N-nucleophiles as well. Furthermore, in the absence of external nucleophiles, in situ-generated 2-iodobenzoate species reacted as nucleophiles, resulting in the formation of allylic esters. Preparation of various allylic ethers was also succeed using EBXs as starting materials via O-VBX formation. The obtained enol ethers **214** could be transformed into α-difunctionalized ketones under oxidative conditions, demonstrating the synthetic utility of the transformation.

#### 4.2.2. Metal-Catalyzed Reactions

**Scheme 44:**
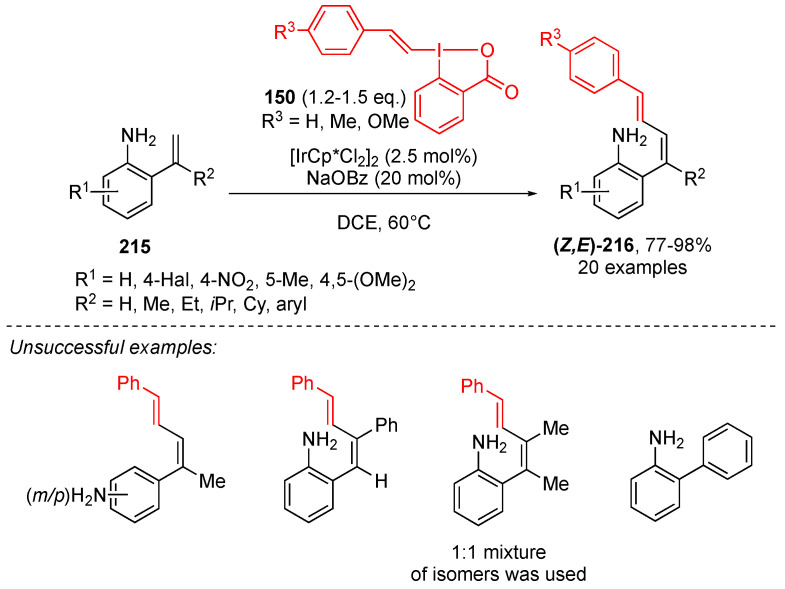
Direct transition-metal catalyzed C-H alkenylation of 2-vinylanilines **215** with the use of C-VBXs **150**.

In 2017, Nachtsheim and co-workers proposed transition metal-catalyzed NH_2_-directed C-H alkenylation of 2-vinylanilines **215** using C-VBXs **150** to synthesize functionalized 1,3-dienes **216** in excellent yields and high (*Z/E*)-stereoselectivity (Scheme 44) [155]. The key deprotonation metalation step was directed by the NH_2_ group, and C-VBXs showed superior reactivity in comparison with non-cyclic vinyliodonium salts.

**Scheme 45:**
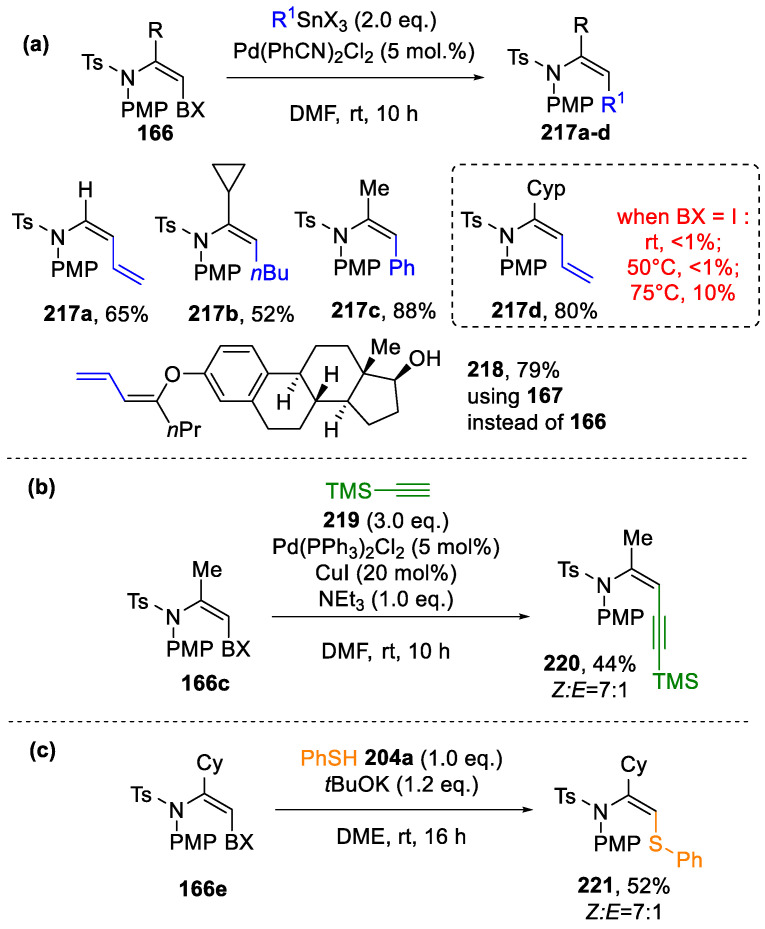
Functionalization of the N- and O-VBXs **166**–**167**. (**a**) Stille coupling; (**b**) Sonogashira coupling; (**c**) thiol addition.

The reported, in Section 4.1.2, N-VBXs **166** have been utilized in the cross-coupling reactions [167]. Pd-catalyzed Stille cross-coupling of vinyl, aryl, and alkyl stannyl reagents and N- and O-VBXs **166–167** to give products **217–218** can be conducted at ambient temperature (Scheme 45a), whereas a similar reaction with simple iodides required heating at 80–120 °C [129,186,187,188]. A direct comparison of the reactivity of monovalent *versus* hypervalent iodine toward cross-coupling was performed on the example of Stille coupling with iodide, but no conversion was observed at room temperature and 50 °C. Less than 10% of the desired product **217d** was observed by ^1^H NMR, together with the significant decomposition of iodide, when the reaction was carried out at 75 °C. This result indicated the high reactivity and synthetic utility of the VBX enamide reagents **166**.

Enyne **220** was then obtained in a 6:1 *Z:E* ratio through a Sonogashira coupling (Scheme 45b) [189,190,191]. Finally, the addition of a strong thiol nucleophile **204a** was possible without a transition metal catalyst to give thioenamide **221** (Scheme 45c) [192,193,194].

**Scheme 46:**
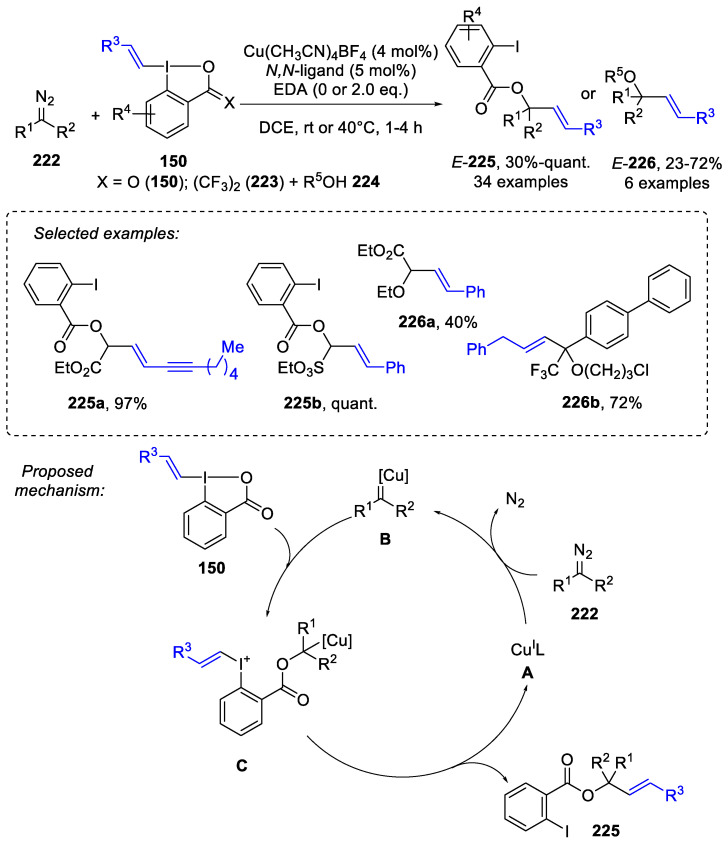
Oxyvinylation of diazo compounds **222** using C-VBXs **150** or **223**. Structures of key intermediates **A**–**C** are shown in the proposed mechanism.

Later, the group of Waser investigated the insertion of VBXs into various diazo compounds **222** under copper catalysis conditions (Scheme 46) [156]. The reaction was proposed to start with the nucleophilic attack of the carboxylate of VBX **150** onto the highly electrophilic copper carbene **B** generated by the reaction of the catalyst **A** with the diazo compound **222**. Vinyl transfer from iodonium intermediate **C** would then give oxyvinylation product **225**. No isomerization of the transferred alkene was observed. The reaction has a good tolerance toward different functional groups in the structure of olefin. The extension of the strategy to a three-component reaction with alcohol nucleophiles **224** and the use of non-nucleophilic benziodoxole-based VBX **223** allowed the synthesis of structurally diverse allylic ethers **226**. All obtained products can be further modified to give important building blocks.

## 5. Conclusions

In summary, the carbon-bonded iodine(III) reagents such as arylbenziodoxoles, ethynylbenziodoxoles, and vinylbenziodoxoles have increased stability in comparison with their acyclic analogs. These reagents can be conveniently prepared from either iodine(I) precursors by one-pot approaches or from other hypervalent iodine compounds. Arylbenziodoxoles, ethynylbenziodoxoles, and vinylbenziodoxoles serve as ‘group transfer reagents’ in a wide range of reactions to afford complex and hard-to-synthesize or/and highly substituted products, which can be modified in further transformations.

Arylbenziodoxoles represent the most stable and readily available class of benziodoxoles; however, their synthetic applications remain limited. The most investigated reaction of arylbenziodoxoles is the benzyne generation under thermal decomposition conditions with subsequent arylation of various nucleophiles. Nucleophilic substitution reactions of arylbenziodoxoles are less investigated and require using transition metal catalysts or heating.

Ethynylbenziodoxoles (EBXs) have been effectively used for the direct alkynylation of diverse nucleophiles, as well as for heterocycle constructions by cascade reactions. These reagents can be applied under various reaction conditions, such as transition metal catalysis, photoredox catalysis, organocatalysis, and transition metal-free reactions to afford a plethora of alkynylated products. The main limitation of these reagents concerns their synthesis, requiring alkynylboronates as starting compounds and purification of the final EBXs.

Vinylbenziodoxoles (VBXs) have attracted recent attention and in many cases were investigated as the products of addition reactions of alkynylbenziodoxoles. VBXs can be used as *E,Z*-selective vinylating reagents for the preparation of various substituted olefins. We believe that in the future, VBXs will find broad application in organic synthesis as convenient and versatile group transfer reagents.

## Figures and Tables

**Table 1:** Preparation of X-VBX [74,140,143,153,167,168,169,170,176].

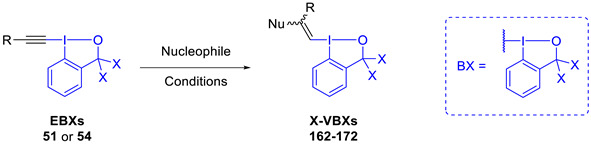				
Entry	Starting EBX	Nucleophile	Conditions	Products and Yields
1 [140]	R = Me; 2X =O	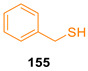	10 mol% 1,1,3,3-tetramethyl guanidine; THF, rt	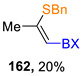
2 [143]	R = Me, *t*Bu, TIPS, Ph, alkyl, incl. functional groups: N_3_, OH, alkene, alkyne, sugar, purine, etc.; 2X = O	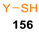 Y = Ph, Bn, proteins, peptides	10 mM Tris pH 8.2 (2% *v*/*v* DMSO); rt	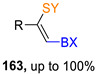
3 [74]	R = H; 2X = O; as chloroform complex	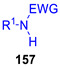 R^1^ = aryl, alkyl, vinyl, 2-furyl-CH_2_-, 2-pyridyl-CH_2_-, MeCO_2_CH_2_-, etc.4 EWG = Ts, Nos, Ms, Tf	10 mol% K_2_CO_3_; IPA or IPA/MeCN (1:1), rt, Ar	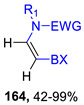
4 [169]	R = H; 2X = O; as acetonitrile complex	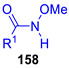 R^1^CO = residues of aromatic and aliphatic carboxylic acid, amino acid, pharmaceutical, and natural products	1M aq. K_2_CO_3_ (20 mol%); DCE or IPA, rt, Ar	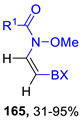
	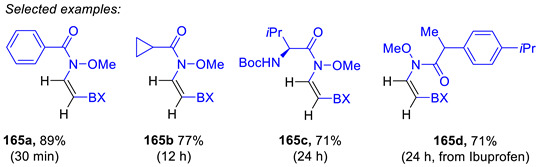			
5 [167]	R = H, alkyl, cycloalkyl, Ph, etc. 2X = O	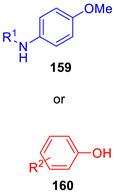 R^1^ = Ts, Ms, Nos; R^2^ = 4-Me, 3,5-Br_2_, 2,3,4,5,6-F_5_	10 mol% Cs_2_CO_3_; EtOH, rt, air	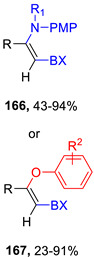
6 [153]	R = aryl, alkyl, alkyne, alkene; X = CF_3_	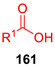 R^1^ = Ph, 4-MeOC_6_H_4_, 4-ClC_6_H_4_, 2-MeC_6_H_4_, Mes, 2-thienyl, 2-furyl, Me, *t*Bu, MeCH(NHBoc)	10 mol% [Pd(2-methylallyl)(OAc)]_2_, cod; *t*BuOMe, rt, 12 h	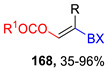
7 [153,176]	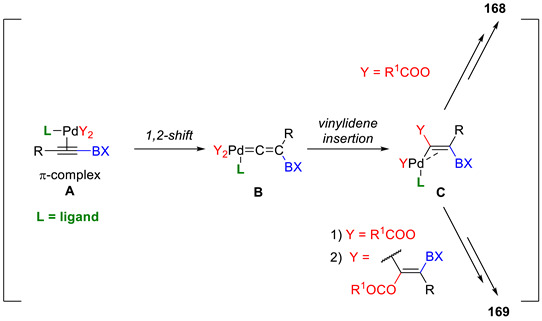			
8 [176]	R = aryl, alkyl, alkyne, alkene; X = CF_3_ (2.4 eq.)	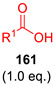 R^1^ = Ph, 4-MeOC_6_H_4_, 4-ClC_6_H_4_, 2-MeC_6_H_4_, 2-thienyl, Me, *t*Bu, MeCH(NHBoc)	10 mol% Pd(OAc)_2_, 15 mol% octahydrophenazine; toluene, rt, 24 h	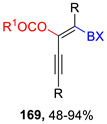
9 [168]	R = H; 2X = O; as acetonitrile complex	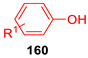 R^1^ = H, EDG, EWG, complex molecules	10 mol% NaHCO_3_; EtOH/H_2_O (9:1 *v*/*v*), rt, dark, 1 h	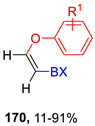
10 [170]	R = aryl, Me, Bu, BnOCH_2_, ethenyl; X = CF_3_	Py·HCl (2.0 eq.) or ICl (2.0 eq.)	EtOAc, 50 °C, 24 h or DCM, rt, 30 min	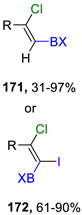

## Data Availability

Data is contained within the article.

## Sample Availability

Samples of the compounds are not available from the authors.
